# Intraspecies Genomic Diversity and Long-Term Persistence of *Bifidobacterium longum*

**DOI:** 10.1371/journal.pone.0135658

**Published:** 2015-08-14

**Authors:** Andrei V. Chaplin, Boris A. Efimov, Vladimir V. Smeianov, Lyudmila I. Kafarskaia, Alla P. Pikina, Andrei N. Shkoporov

**Affiliations:** 1 Microbiology and Virology Department, Pirogov Russian National Research Medical University, Moscow, Russia; 2 Department of Natural Sciences, Medical Institute, North Caucasus State Academy for Humanities and Technologies, Cherkessk, Russia; University of Ulm, GERMANY

## Abstract

Members of genus *Bifidobacterium* are Gram-positive bacteria, representing a large part of the human infant microbiota and moderately common in adults. However, our knowledge about their diversity, intraspecific phylogeny and long-term persistence in humans is still limited. *Bifidobacterium longum* is generally considered to be the most common and prevalent species in the intestinal microbiota. In this work we studied whole genome sequences of 28 strains of *B*. *longum*, including 8 sequences described in this paper. Part of these strains were isolated from healthy children during a long observation period (up to 10 years between isolation from the same patient). The three known subspecies (*longum*, *infantis* and *suis*) could be clearly divided using sequence-based phylogenetic methods, gene content and the average nucleotide identity. The profiles of glycoside hydrolase genes reflected the different ecological specializations of these three subspecies. The high impact of horizontal gene transfer on genomic diversity was observed, which is possibly due to a large number of prophages and rapidly spreading plasmids. The pan-genome characteristics of the subspecies *longum* corresponded to the open pan-genome model. While the major part of the strain-specific genetic loci represented transposons and phage-derived regions, a large number of cell envelope synthesis genes were also observed within this category, representing high variability of cell surface molecules. We observed the cases of isolation of high genetically similar strains of *B*. *longum* from the same patients after long periods of time, however, we didn’t succeed in the isolation of genetically identical bacteria: a fact, reflecting the high plasticity of microbiota in children.

## Introduction

The genus *Bifidobacterium* comprises Gram-positive high G+C rods belonging to the phylum *Actinobacteria* [1]. It is commonly believed that bifidobacteria predominate in microbiota of breast-fed and formula-fed infants [2]. It is also estimated that bifidobacteria constitute nearly 11% of intestinal microbiota in children aged 1–4 years and account for a significant portion of the gut microbial consortium in adults [2,3]. Intestinal bifidobacteria have evolved to specialize in the fermentation of a variety of carbohydrates that are not digested by a host macroorganism using a complex metabolic network including unique galacto-N-biose/lacto-N-biose and fructose-6-phospate phosphoketolase pathways [4]. Consecutively, more than 8% of the identified genes in most studied bifidobacterial genomes are predicted to participate in carbohydrate transport and metabolism [5]. Bifidobacteria are widely used as probiotics as they are considered to confer health benefits to their human hosts. In particular, bifidobacteria produce water-soluble vitamins [6] that can be absorbed by the host [7]. Immunoregulatory properties of bifidobacteria, such as the ability to suppress the inflammatory responses, are well-documented by *in vitro* and *in vivo* studies [8,9]. The spectra of specific cytokines induced or suppressed by individual *Bifidobacterium* species or strain can vary greatly [10–12]. Certain strains of bifidobacteria are known to produce bacteriocins active against both Gram-positive and Gram-negative bacteria, including pathogens [13]. Additionally, such biological properties as bile and acid resistance that are considered to be of importance for probiotic activity also vary significantly among bifidobacterial species as well as between different strains within a single species [14].

While the species composition of bifidobacterial population in human intestinal tract undergoes significant changes with the age, *Bifidobacterium longum* is generally considered to be the most common and prevalent species found in this habitat both in infants and adults [2,15,16]. The species of *B*. *longum* comprises three known subspecies: *longum*, *infantis* and *suis* [17]. The former two subspecies are commonly found in human intestinal microbiota. However, while *B*. *longum* subsp. *longum* is widely distributed in both adults and infants, *B*. *longum* subsp. *infantis* appears to be specialized in the fermentation of human milk oligosaccharides and thus can be detected in infants but not in adults [18,19]. The subspecies of *B*. *longum* subsp. *suis* is considered to be characteristic to porcine intestinal microbiota, and is closely related to *B*. *longum* subsp. *longum* [20].

Intraspecies genomic diversity of *B*. *longum* has been studied in DNA-DNA hybridization studies [21] and by comparing a limited number of complete genome sequences [22,23]. A significant role of horizontal gene transfer (HGT) in the evolution of *B*.*longum* was predicted based on the first complete genome sequence of this species [24]. Recently, a number of additional new whole genome sequencing (WGS) projects of *B*. *longum* strains of human origin have been completed [23,25,26] thus allowing for the comprehensive comparative genomics studies of this species. Such an investigation would enable, in particular, the roles of HGT and other mechanisms in the generation of genomic diversity of *B*. *longum* to be thoroughly evaluated.

The mechanisms of establishment and persistence of bifidobacterial strains within the human intestinal microbial community are not well understood. The DNA fingerprinting-based study of the intestinal bifidobacteria demonstrated major changes in strain composition in children during a 5-year period, but at least in some cases the dominant strain of *B*. *longum* appeared to remain unchanged [27]. In our previous study, we sequenced genomes of these persisting strains and confirmed their close relatedness but not complete identity [28].

In the present study, we extended the genetic knowledge of the intraspecies genomic diversity of *B*. *longum* residing in human gut by performing comparative analysis of 28 genomes, including the sequences of strains isolated from the same individuals during a longitudinal observational study.

## Materials and Methods

### Ethics Statement

The study was approved by the Ethics Committee of Pirogov Russian National Research Medical University. Written informed consent was obtained from parents of each subject.

### Strain isolation and genome sequencing

The strains selected for WGS in this study were isolated at several sampling points over an 11-year time period from feces of three healthy children. Homogenized feces were serially diluted in freshly autoclaved 0.9% NaCl and plated onto Bifidobacterium-agar (Himedia Labs, India). Petri dishes were incubated at 37°C for 48h in anaerobic jars (Schuett Biotech, Germany) filled with anaerobic gas mixture (85% N_2_, 10% H_2_, 5% CO_2_). The morphologically distinct types of bacterial colonies were subcultured and assessed by microscopic Gram stain examination and potential bifidobacterial isolates were selected for the study. The cultures were preserved by freeze-drying of suspensions in 10% sucrose/1% gelatin (w/v) solution. The preliminary designation of isolates to *B*. *longum* subsp. *longum* and *B*. *longum* subsp. *infantis* subspecies was performed using PCR with 16S rRNA gene-targeted species-specific primer sets [29]. DNA typing of bifidobacteria was performed using PAGE analysis of PCR-amplified variable number tandem repeat (VNTR) loci ##12, 23 and 25 essentially as described by Matamoros *et al*. [30] to discriminate between different strains of the same subspecies isolated at a particular sampling point from the individuals.

Вifidobacterial DNA was extracted by the method of Stahl [31] with minor modifications. Briefly, 4 ml of overnight cultures were centrifuged, washed once with TES buffer (50 mM NaCl, 100 mM TrisHCl, pH 8.0, and 70 mM EDTA) and resuspended in 250 ml of the same buffer supplemented with 25% sucrose, 30 mg/ml lysozyme, and 70 U/ml mutanolysin, and incubated at 42 C for 1 h. The subsequent steps were identical to the original protocol. Sequencing of the genomic DNA was performed at Genotek LLC (Russia) on Illumina HiSeq 2000 platform using TruSeq HT V3 kits. Reads were *de novo* assembled with CLC Genomics Workbench, the coverage varied from 1059x to 3037x.

The orthologous loci of CRISPR-1 system were amplified using primers 5’-CCCTATGGATGGTGGAAATCAG-3’ and 5’-CCCTATGGATGGTGGAAATCAG-3’. Polymerase chain reactions were performed in a total volume of 20 μl containing 1× PCR buffer, 0.25 μmol l^−1^ of each primer, 0.15 mmol l^−1^ of each dNTP, and 1 U of Taq polymerase (SibEnzyme, Russia). Amplification was performed using 30 cycles of (95°C, 30 s; 60°C; 30 s, 72°C; 3 min). For amplification of CRISPR-2 system loci we used nested PCR. The following external primers were used: 5'-GTTGCTCGACATGGGATATGG-3' and 5'-TGGATCTGGTACAGGGTGAC-3'. Sequences of the internal primers were as follows: 5'-GTATACGCCTACGCAATCGG-3' and 5'-AACATCCGCCGATAAACAGTC-3'. Reaction was performed using 15 cycles at the first stage of nested PCR and 15 cycles at the second stage under the same conditions as for locus CRISPR-1. Sanger sequencing of PCR products, obtained from strains 44B, 1-6B, 35B and 2-2B, was performed by primer walking at Evrogen JSC (Russia). Integration of streptococcal mobile genetic element into the genome of *B*. *longum* subsp. *infantis* EK3 was confirmed using PCR with primers 5’-CCACTTCTCCAGCGGATGTT—3’ and 5’- TGACCGCAAGAAGGTGTCTC-3’. Amplification was performed using 30 cycles of (95°C, 30 s; 58°C; 30 s, 72°C; 2 min).

### Sequence analysis

All genome sequences analyzed in the study were uniformly (re)annotated using RAST with default settings and with "Fix frameshifts" parameter, which enables search and joining of genes fragmented by frameshift sequencing errors or mutations by comparing with the template genes in the nearest neighbors [32]. Whole genome alignment was performed using progressive Mauve [33]. The Average Nucleotide Identity (ANI) between the strains was calculated using JSpecies [34]. IS elements were annotated using ISsaga in the automatic mode [35]. Restriction-modification systems were annotated using blastp on REBASE database [36,37].

For phylogenetic inference we selected sequences of 43 presumptive housekeeping genes from the strains of *B*. *longum* as well as from *B*. *breve* UCC2003 [38] (see S1 Table). All of these were present in all of the genomic sequences studied and did not contain any undefined positions. The genes were chosen to be distanced from each other for more than 10 kb in the genome of a reference strain JCM 1217^T^ to avoid possible sequence changes in two or more genes as the result of a single recombination event. In case several housekeeping genes were closely located the largest of them was chosen for analysis.

Gene sequences were aligned using MUSCLE [39] followed by manual curation. Phylogenetic tree was obtained using concatenated nucleotide alignments generated with Neighbor Joining algorithm. To detect recombination event(s) we used PhiPack [40] indirect recombination tests: NSS, Max χ^2^, PHI test using normal probability distribution and PHI test with 100 000 permutations of nucleotide alignments. Phylogenetic trees corrected for recombination events were reconstructed using ClonalFrame software with 100 000 iterations [41]. Three replicate runs were done to assess convergence of results [42,43]. This method is computationally intensive so no bootstrapping was performed. Alignment and phylogenetic inference of 16S rRNA genes and pKJ36/pB44 family plasmids were also performed using MUSCLE and Neighbor Joining algorithms.

Orthologs search was performed using OrthoMCL using parameters recommended by software developers: e-value cut-off 1e-5 and MCL inflation index I = 1.5 [44]. To evaluate the number of homoplasies in a dataset describing presence or absence of each ortholog group within the genomes of *B*. *longum*, we calculated pairwise compatibility score for each pair of ortholog groups as described in [40]. The core-and pan-genome size dynamics of *B*. *longum* subsp. *longum* were inferred using 10 000 random permutations of sequential inclusion of bacterial strains. The type of pan-genome was determined using a power law model described by Tettelin *et al*. [45] using mean values as well as medians of the number of ortholog groups. To explore the variation of the power law coefficient 50 000 "delete-half" subsamples were made and the coefficient was inferred using 1000 random permutations of sequential inclusion of strains within each subsample The ortholog groups within the pan-genome were divided into core, moderately distributed, and rare genes, based on the presence of orthologs in all, in several but not all, and in only one strain, respectively. Functional grouping of genes was made using RPS-BLAST on COG database [46], conservative domain search was made using Pfam database [47]. The comparison of functional group frequencies among genes of known functions in different parts of pan-genome was performed using Fisher's exact test with Benjamini-Hochberg controlling procedure.

Glycoside hydrolases in the genome sequences of *B*. *longum* were annotated using CAZymes Analysis Toolkit [48]. The families 23, 25, 73 and 103, comprising peptidoglycan hydrolases or peptidoglycan lyases, were excluded from the analysis since their abundances would more likely reflect the number of prophages in a genome rather than metabolic capabilities of a strain. The mean-centered scaled quantities of members of each remaining family were used for principal component analysis (PCA). Non-uniformity of distribution of glycoside hydrolase families between subspecies was estimated using Kruskal-Wallis test with Holm-Bonferroni correction.

Phage-related sequences in the genomes sequences were detected using PHAST [49] with manual curation of the results. The search for plasmids was performed based on homology with known plasmid sequences and the coverage of contigs. The circularization of plasmid sequences was made using the overlapping ends of contigs acquired by mapping of reads using Bowtie 2 [50].

Putative CRISPR loci were identified using CRISPRFinder [51] and flanking sequences were checked for the presence of *cas* genes. The protospacers were located by running BLASTn searches within *nr/nt* and *wgs* databases [37].

## Results and Discussion

### Strain isolation and selection

**Fig 1:**
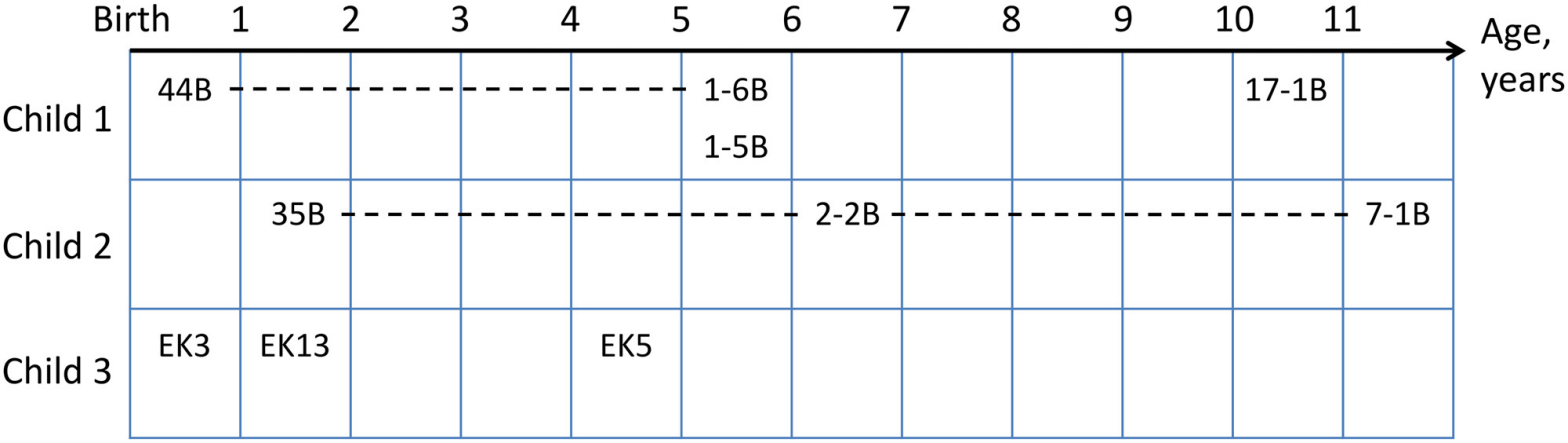
Strains, isolated from the same children in different time points. Horizontal axis represent the age of the subject, rows represent the children, labels in the cells correspond to the isolated strains. Dotted lines connect similar strains.

Most of the strains (10 out of 12, see Fig 1) sequenced for this study were isolated as numerically predominant bifidobacteria at several sampling time points during an extended, 11-year long, observational study of intestinal microbiota in three healthy children. For all strains identified as *B*. *longum* we performed VNTR analysis as a preliminary measure of their genetic similarity. Numerically predominant strains isolated from the children at every sampling point and showing unique VNTR patterns or forming similarity groups with other strains were selected for further studies.

Two distinct groups of *B*. *longum* strains isolated from two children subjects could be established according to VNTR analysis (Fig 1). The first group was formed by strain 44B (isolated from Child 1 at the age of 9 months) and strain 1-6B (isolated from the same child at the age of 5 years). The second group included the strains from Child 2: 35B (isolated at age of 11 months), 2-2B (isolated at the age of 6 years) and 7-1B (isolated at age of 11 years). The similar strains within these groups could be the derivates or each other or represent a distinct phylogenetic lineages. Four of these strains (44B, 1-6B, 35B and 2-2B) were described previously [27,28] and their draft WGSs were reported.

To evaluate general genetic diversity of *B*. *longum* species we also sequenced the genomes of several additional strains, namely strains 1-5B and 17-1B that were also isolated from a Child 1, but could not be grouped by VNTR analysis. We also sequenced unrelated strains EK3, EK5 and EK13 from a Child 3, and two additional unrelated strains of human origin, described in previous works: 72B and VMKB44, both isolated from child feces [52,53]. In total, the draft genome sequencing of eight *B*. *longum* strains of human origin was performed, and four 4 additional strains from the same cohort were sequenced earlier (44B, 1-6B, 35B, and 2-2B) [28]. Among these 12 strains 11 belonged to *B*. *longum* subsp. *longum*, while the strain EK3 belonged to *B*. *longum* subsp. *infantis*. All strains underwent 4–5 passages between isolation and sequencing, except for VMKB44 which undergone considerably more *in vitro* transfers. For the purpose of comparative genomic analysis a number of publicly available (as per September, 2014) complete and draft genomic sequences of *B*. *longum* strains were included in the analysis. Although the low quality sequences can bias the results, the higher the number of included genomes could allow for more representative view on the species genomics. All sequences were uniformly reannotated using RAST with “Fix frameshifts” parameter, which performs homology-based search of gene fragments to minimize negative effect of possible sequencing errors as well as to find recent pseudogenes. Neither draft assemblies from metagenomes, nor the genomic sequences from several derivatives from a single strain were included. In total, 20 genomic sequences available from the other studies were included, so that the final number of genomes analyzed in this study was 28: 8 complete and 20 draft (Table 1, see S2 Table for details on the genome sequencing of the strains). Most of the analyzed strains with known source of isolation were obtained from the human feces except for a strain LMG 21814^T^ (isolated from pig feces) and strain AGR2137 (isolated from cow rumen).

**Table 1:** General properties of the *B*. *longum* genomes analyzed in this study.

Strain name	GenBank accession number	Reference	Genome sequence status	Sequence length, Mbp	Subspecies identified in this study
ATCC 15697^T^	NC_011593	[38]	Complete	2.83	*infantis*
EK3	JNWC00000000	this study	45 contigs	2.56	*infantis*
AGR2137	ATWX00000000	-	49 contigs	2.27	*suis*
JDM301	NC_014169	[54]	Complete	2.48	*suis*
LMG 21814^T^	JGZA00000000	[55]	36 contigs	2.34	*suis*
12_1_47BFAA	NZ_ADCN00000000	-	86 contigs	2.40	*longum*
157F	NC_015052	[56]	Complete	2.40	*longum*
1-5B	JNVX00000000	this study	28 contigs	2.37	*longum*
17-1B	JNVZ00000000	this study	23 contigs	2.47	*longum*
1-6B	AJTF00000000	[28]	171 contigs	2.69	*longum*
2-2B	AJTJ00000000	[28]	141 contigs	2.63	*longum*
35B	AJTI00000000	[28]	131 contigs	2.51	*longum*
44B	AJTM00000000	[28]	62 contigs	2.56	*longum*
72B	JNWA00000000	this study	39 contigs	2.37	*longum*
7-1B	JNVY00000000	this study	37 contigs	2.41	*longum*
ATCC 55813	ACHI00000000	[57]	140 contigs	2.37	*longum*
BBMN68	NC_014656	[26]	Complete	2.27	*longum*
CCUG 52486	NZ_ABQQ00000000	-	55 contigs	2.45	*longum*
CECT 7347	NZ_CALH00000000	-	128 contigs	2.33	*longum*
DJO10A	NC_010816	[23]	Complete	2.38	*longum*
E18	AUYD00000000	[58]	7 contigs	2.37	*longum*
F8	NC_021008	-	21 contigs	2.37	*longum*
EK5	JNWC00000000	this study	35 contigs	2.23	*longum*
EK13	JNWD00000000	this study	48 contigs	2.47	*longum*
JCM 1217^T^	NC_015067	[56]	Complete	2.39	*longum*
KACC 91563	NC_017221	[25]	Complete	2.39	*longum*
NCC2705	NC_004307	[24]	Complete	2.26	*longum*
VMKB44	JRWN00000000	this study	36 contigs	2.51	*longum*

A recent study showed the presence of conjugative megaplasmids in *B*. *longum* subsp. *longum* strains 44B, 1-6B and 2-2B [59]. However, due to the draft status of these genomic sequences we couldn't accurately determine what contigs in the assemblies correspond to the plasmid. The same situation potentially could be present in the other draft genome sequences of *B*. *longum*. So, in our analysis we used the term 'genome' in a broad sense, including not only the chromosome of the strain, but also possible episomal elements.

### Intraspecies phylogeny of *B*. *longum*

**Fig 2:**
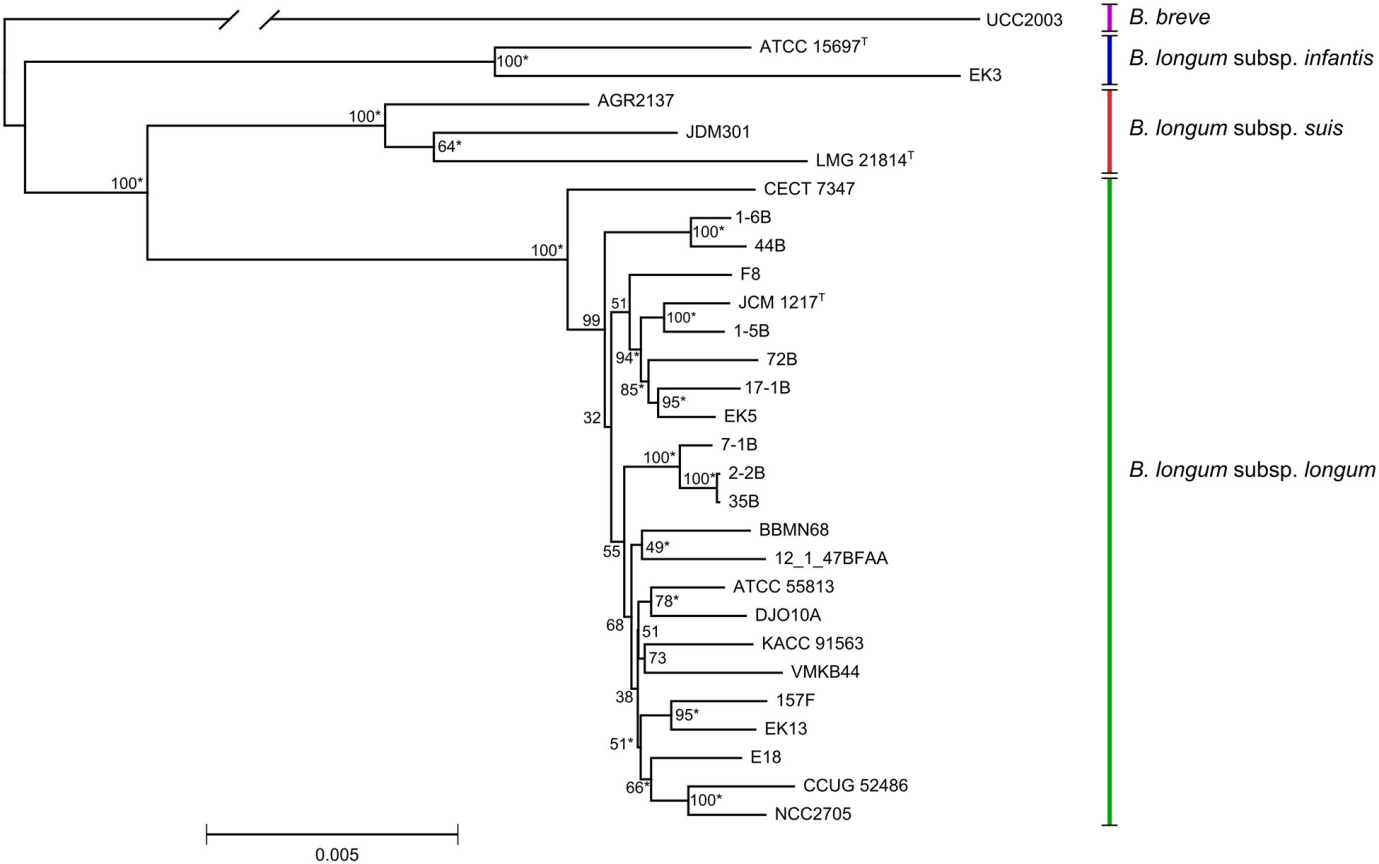
Neighbor-joining phylogenetic tree of *B*. *longum* based on concatenated sequences of 43 housekeeping genes. *Bifidobacterium breve* strain UCC2003 was used as an outgroup (branch length is given out of scale). Clades confirmed by ClonalFrame analysis are marked with asterisk (*), numbers in nodes represent bootstrap confidence levels.

Using the procedure described in Materials and methods section, we selected a set of 43 presumptive housekeeping genes (S1 Table) that were present in all studied genomes of *B*. *longum* and their homologous counterparts in *B*. *breve* UCC2003 [38], performed multiple alignments of concatenated sequences and a phylogenetic analysis using Neighbor joining algorithm. As a result, we managed to cluster the strains into the three major groups (Fig 2). Each of these groups contained a type strain for one of the three subspecies of *B*. *longum* (JCM 1217^T^ for the *B*. *longum* susbp. *longum*, ATCC 15697^T^ for the *B*. *longum* subsp. *infantis* and LMG 21814^T^ for the *B*. *longum* subsp. *suis*). Hence, we associated those three clusters with the three subspecies. Importantly, the phylogenetic clustering based on the analysis of gene content and average nucleotide identity (ANI, S1 Fig, described below) showed identical results in the subspecies identification. However, several conflicts between our clustering and the original strain descriptions were noted: strain JDM301 which was initially described as *B*. *longum* subsp. *longum* falls into *B*. *longum* subsp. *suis* group, while *B longum* stains 157F, ATCC 55813 and CCUG 52486 initially described as *B*. *longum* susbp. *infantis* clearly belong to *B*. *longum* subsp. *longum*. The latter two conflicts were also previously noted by LoCascio *et al*. [18], suggesting the strains had been mis-identified in the original studies.

The indirect recombination tests (NSS, Max χ^2^, PHI test using normal probability distribution, PHI test with 100000 permutations), of PhiPack software package [40] provided the p-value of 0 on the concatenated sequences of the chosen housekeeping genes, indicating the presence of recombination events. Accordingly, while curating the alignments manually we observed numerous events of HGT between the three subspecies of *B*. *longum*, that supposedly occurred through homologous recombination with gene conversion. Moreover, we found transfers of gene fragments from the most closely related species, *Bifidobacterium breve*, which formed the sequence of the gene coding for glucosamine-fructose-6-phosphate aminotransferase (*glmS*, NZ_CALH01000030.1) in *B*. *longum* subsp. *longum* CECT 7347 (S1 Fig), and the gene encoding UDP-N-acetylglucosamine enolpyruvyl transferase (*murA*, JGZA01000026.1) in *B*. *longum* subsp. *suis* LMG 21814^T^.

HGT may significantly affect the phylogeny reconstruction leading to the distorted tree topology, increased branch lengths and general low robustness of the phylogenetic inference. Therefore, we employed the ClonalFrame software that implements a Bayesian phylogenetic approach to acquire a phylogeny reconstruction unaffected by recombinations using the same set of genes from the 28 strains of *B*. *longum* with *B*. *breve* UCC2003 used as an outgroup. (Fig 2). We also inferred the relative impact of mutations and HGT on genomic variability in *B*. *longum* using the ClonalFrame software on the dataset, containing only the genes of *B*. *longum* strains. The calculated ratio of recombination events to point mutations was 0.047, and the ratio of nucleotide substitutions by recombination to point mutations was 0.72.

Our study confirmed that the 16S rRNA sequence is informative enough for the discrimination between *B*. *longum* subsp. *infantis* and *B*. *longum* subsp. *longum* [29]. The most pronounced nucleotide differences between the subspecies of *B*. *longum* are located within the V6 region of 16S rRNA gene. However, the discrimination between human-associated subspecies and *B*. *longum* subsp. *suis* was shown to be less robust. Two of the three *B*. *longum* subsp. *suis* genome sequences available contain assembled 16S rRNA gene sequences: the *B*. *longum* subsp. *suis* LMG 21814^T^ 16S rRNA gene is similar to 16S rRNA genes of *B*. *longum* subsp. *longum* (99.5%-99.7% identity), while the 16S rRNA gene of strain *B*. *longum* subsp. *suis* JDM301 is identical with the 16S gene of *B*. *longum* subsp. *infantis* ATCC 15697^T^. Further phylogenetic analysis confirmed clustering of *B*. *longum* subsp. *suis* JDM301 with *B*. *longum* subsp. *infantis* based on 16S rRNA sequences with high bootstrap confidence level (Fig A in S2 Fig). This observation could hardly be explained by phylogenetic noise, since it would take 7 random nucleotide substitutions to make one sequence fully identical to the other related sequence (Fig B in S2 Fig). One possible explanation is the paraphyly of *B*. *longum* subsp. *suis*, which however contradicts to the topology of tree based on housekeeping genes. Thus, the most likely explanation of the observed data is intraspecific HGT of 16S rRNA gene after the divergence of *B*. *longum* subspecies. a rare, but possible event.

As predicted by VNTR typing the strains 1-6B and 44B (isolated from child 1) formed a very tight cluster (99.8% identity). A comparable degree of identity (99,7%) was observed between the strains 2-2B, 35B and 7-1B, isolated from child 2. However, none of the strains in these groups were found to be completely identical. While the most closely related strains, 2-2B and 35B, demonstrated a very high sequence identity of the chosen housekeeping genes (only 6 out of 65310 bp used for the alignment were different, and 4 of these 6 differences were found to be unambigous using manual curation of raw reads, i.e. the identity percentage was 99,99%), the distances between other strains isolated from the same children at different time points (Fig 1) were found to be several folds larger. For example, we found major differences in the sequences of the genes coding for aspartyl-tRNAsynthetase (*aspS*), UDP-N-acetylmuramoylalanyl-D-glutamate 2,6-diaminopimelate ligase (*murE*), prolyl-tRNA synthase (*proS*), adenylosuccinate lyase (*purB*), and RNA polymerase σ-subunit (*rpoD*) between the strains 1-6B and 44B that indicate possible recombination events after the divergence of the lineages leading to these two strains.

To make the clustering based on an alternative approach we also measured the whole-genome average nucleotide identity (ANI) between all pairs of the strains (S3 Fig). It was calculated with Jspecies software [34] using pipeline described by Goris *et al*: a genome sequence from one of the strains in a pair was cut into 1020 bp fragments, which were searched in the other genome using bastn; the ANI between the query genome and the reference genome was calculated as the mean identity of all blastn matches that showed more than 30 % overall sequence identity (recalculated to an identity along the entire sequence) over an alignable region of at least 70% of their length [60]. Thus, this calculation included loci changed by mutations or transferred via homologous recombination, but not those acquired by other mechanisms of HGT. The ANI-based clustering of strains supported the division of *B*. *longum* into three distinct subspecies, and found the same strains as in the housekeeping gene clustering to be highly similar. Thus the ANI between the two strains of *B*. *longum* subsp. *infantis* was 98.28%, the mean ANI between the strains of subsp. *suis* was 98.35%, and the mean ANI between the strains of *B*. *longum* subsp. *longum* was 98,89% (98,88% after the exclusion of highly similar strains 1-6B, 44B, 2-2B, 25B and 7-1B). Interestingly, the identity between the *B*. *longum* subsp. *longum* and *B*. *longum* subsp. *infantis* varied between 95% and 96% (mean ANI 95,66%), approaching the threshold for classification prokaryotic organisms in the same species. At the same time, identities between other pairs of subspecies were higher (mean ANI 96,37% between subspecies *infantis* and *suis*, 96,99% between subspecies *longum* and *suis*).

### General genome comparison

To determine the general characteristics of *B*. *longum* genome we analyzed all 28 available draft and completed genomic sequences. For the draft sequences we estimated the genome size as a sum of all contigs lengths, which could be smaller than the real genome size due to contig copy number variation. We found that genome size was unimodally distributed with median value of 2.39Mbp (S4 Fig). The largest genome (2,83Mbp) among the strains analyzed in this study belonged to the strain ATCC 15697^T^, the type strain of *Bifidobacterium longum* subsp. *infantis*. Generally, the strains of subsp. *infantis* had larger genomes than the strains of subsp. *longum* (Mann–Whitney *U* test, p = 0.02). No significant difference in the genome size between subsp. *suis* and subsp. *longum* was detected.

An early work on comparative genomics of *B*. *longum* suggested the attenuation during multiple culture transfers on artificial media as the major source of the differences in genome size between the different strains of *B*. *longum* [23]. However, the draft genomes of *B*. *longum* subsp. *longum*, sequenced and assembled in this study using the identical protocol, showed high diversity of summed sequence lengths ranging from 2.25 to 2.51 Mpb. Moreover, the largest draft genome sequence belonged to the strain VMKB44 that had had the longest history of culture transfers within this set. Thus we speculate that natural variations have higher impact on genome size in *B*. *longum* than the culturing history of the strain.

**Fig 3:**
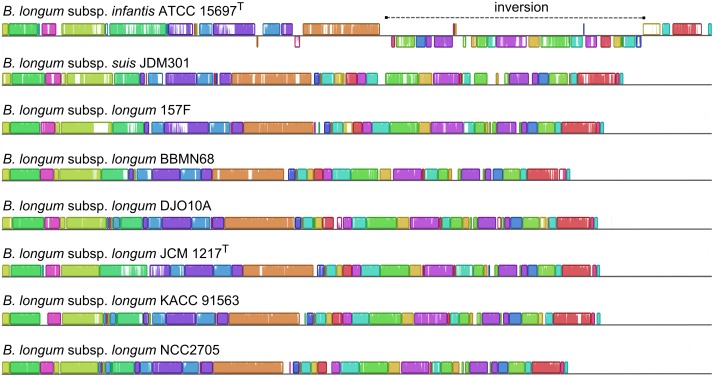
Whole genome alignment of complete genome sequences using Mauve. Colored boxes, linear collinear blocks (LCB). White gaps, insertions and deletions. Position atop or below the horizontal line represents the direction of LCB.

The mean GC-content of *B*. *longum* genome was found to be 59.97±0.23%. In most strains it varied between 59.63% and 60.33%, with the exception of strain B. *longum* subsp. *infantis* EK3, whose GC-content was found to be 59.38%. Although, this number was calculated from draft genome data, he real chromosome of this strain could have even lower proportion of GC pairs. One contig from this assembly (JNWB01000008.1, 3733 bp) with a high sequence coverage corresponding to roughly 20 copies in genome has a GC-content of 36.71% and represents a putative mobile element, possibly acquired from a member of genus *Streptococcus*. Using the analysis of raw paired-end reads we found that different copies of this element are adjacent with different contigs of the assembled sequence. As an example, one of the copies was localized near the bifidobacterial prolyl-tRNA synthase gene (*proS*). Using PCR with the primers specific to the mobile element and *proS* gene we confirmed that this element is integrated in the genome of strain EK3 rather than being a contamination of the DNA sample. Analysis of this contig using blastx shows, that it contains an open reading frame encoding mercuric ion reductase. However, it is unknown whether this streptococcal gene is actually expressed in *B*. *longum*. The structural genome characteristics of *B*. *longum* species were determined using the eight completed genomes included in this study. Whole genome alignment showed high level of synteny with only one large rearrangement observed in the genome of *B*. *longum* subsp. *infantis* ATCC 15697^T^ (Fig 3). Three loci, containing rRNA genes, were found to be present in each available completed genome sequences (S5 Fig). However, while in some strains each locus contained only one rRNA operon, in others one of these loci contained two operons, forming a tandem repeat. Accordingly, the total number of rRNA operons varied from 3 to 4. The number of tRNA genes among most strains with complete genome sequences varied between 55 and 60, except for JCM 1217^T^ and ATCC 15697^T^, which were found to harbor 76 and 79 tRNA genes, respectively.

Mobile genetic elements play an important role in the generation of genomic diversity and horizontal gene transfer in bacteria [61].Using ISsaga pipeline of annotation [35] based on the ISfinder database [62], we performed an automated prediction of IS elements, the simplest prokaryotic mobile genetic elements, in eight completed genomes of *B*. *longum*. While the genomes of subspecies *longum* and *suis* contained 28–40 unique IS elements, the larger genome of B. *longum* subsp. *infantis* ATCC 15697 harbored 61 loci. Some of the IS families were found in all completed genomes of *B*. *longum* (IS3, ISL3, IS21, IS30, IS21), but most of them were present only in a few or even in a single completed genome. The total number of IS elements per genome in *B*. *longum* varied between 42 and 90 (Table 2), which is appreciably higher than previously found in *B*. *breve* by Bottacini *et al*. (from 12 to 54) [63]. We have also performed a search in *B*. *breve* using the same strain set as in the cited work and detected from 22 to 53 IS-elements, significantly less than found in *B*. *longum* (Mann–Whitney *U* test, p = 0.005). This observation is in accordance with the previous report [6] that found *B*. *longum* species, especially the *B*. *longum* subsp. *infantis*, to harbor the largest and the most diverse set of IS elements.

**Table 2:** Total numbers of IS elements of different families in the complete *B*. *longum* genome sequences.

	ATCC 15697	JDM301	157F	BBMN 68	DJO10A	JCM 1217	KACC 91563	NCC2705
IS1595_ssgr_ISPna2	0	0	0	2	0	0	0	0
IS200_IS605_ssgr_IS1341	5	0	1	0	0	2	0	0
ISL3	5	7	6	6	8	8	4	9
IS5_ssgr_IS427	2	0	0	0	0	0	2	0
IS607	2	2	0	0	2	1	0	1
IS256	12	5	2	2	3	1	11	6
IS256_ssgr_IS1249	7	9	0	0	4	0	4	0
ISNCY	0	1	0	0	0	0	0	0
IS66	0	0	1	2	2	1	1	0
IS30	21	5	19	12	10	20	9	4
IS3_ssgr_IS150	15	11	8	8	10	5	5	6
ISNCY_ssgr_ISLbi1	0	2	0	0	0	0	3	0
IS200_IS605	1	0	0	0	0	0	0	0
IS21	8	8	22	16	14	8	10	8
IS3_ssgr_IS51	0	0	0	1	0	0	0	0
IS3	3	14	6	1	4	6	4	8
IS110	9	0	0	0	0	0	0	0
Total	90	64	65	50	57	52	53	42

Bacteriophages represent another important driver of intraspecies variability in bacteria. While no bifidobacterial phages were isolated in culture, a large number of prophage-like elements were detected, and some of them were found to be inducible at the transcription level [6,64]. Nine putative prophage-like elements were found in *B*. *longum* by Ventura *et al*. [64]. A preliminary analysis of other genomes for the presence of prophage elements conducted in this study revealed much more diverse prophage-like sequences. For example, the genome of the strain 157F contains a 62 kb prophage (BLIF_0804-BLIF_0890) that demonstrates no close homology within the other known genomic sequences. Another examples of the prophage without whole-sequence homologs is a 34 kb putative prophage in *B*. *longum* 44B (HMPREF1312_1242-HMPREF1312_1295) and a 36 kb putative prophage in *B*. *longum* VMKB44 (NL89_04820—NL89_05060). All strains isolated from Child 2 (i.e. 35B, 2-2B and 7-1B) were found to contain a 38 kb prophage (BL71B_01030-BL71B_01340) that was 99% identical within these strains and partially homologous to the prophages from strains EK13 and CCUG 52486.

### Gene content of *B*. *longum*

**Fig 4:**
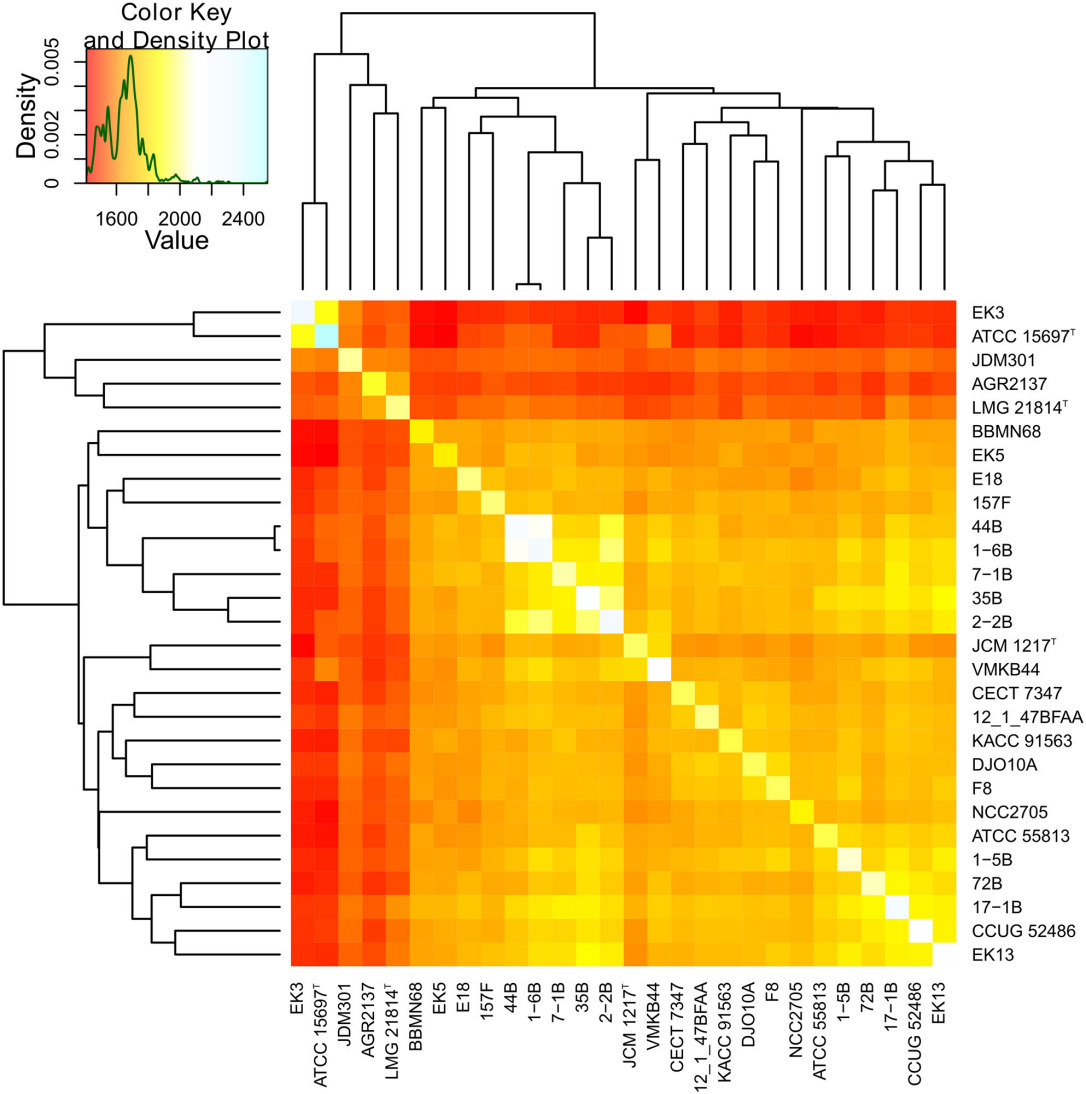
Pairwise comparison of ortholog groups content in *B*. *longum* strains. Heatmap represents the number of shared orthologs between strains, the tree was inferred by complete linkage hierarchical clustering.

To analyze the gene content of *B*. *longum* strains we performed standardized re-annotation of all 28 genomes and orthologs clustering. Totally 5324 orthologous groups were identified, including 287 groups that contain multiple paralogous genes at least within one of the genomes and 1527 groups that are represented by a single member in only one genome (singletons). The number of orthologous groups in an average strain was accounted to be 2049±163, less than a half of the total number of orthologous groups that were found in the clade. To construct a similarity heatmap we performed pairwise comparisons of the numbers of matching conserved orthologous groups between all sequenced genomes (Fig 4). Using the hierarchical clustering based on these data, we obtained the same separation *B*. *longum* strains into subspecies clusters as described above for ANI and house-keeping genes clustering. However, in contrast to the phylogeny inferred from the house-keeping genes data, *B*. *longum* subsp. *suis* was found to be closer to the subsp. *infantis*, than to subsp. *longum*, sharing slightly more ortholog groups with it (1554 vs 1536 on average).

A total of 1198 orthologous groups were present in all strains of *B*. *longum*, thus forming the core genome of the species. We also calculated the 'core additions' for each of the subspecies, i.e. orthologous groups conserved in all genomes of a particular subspecies, but absent from all other genomes. For *B*. *longum* subsp. *infantis* the 'core addition' was found to contain 175 orthologous groups. By contrast, the mean value of 'core addition' orthologous groups calculated for all other random divisions of strains into two groups of 2 and 26 randomly picked genomes was found to be 1.018. Such a large number of orthologous groups, found exclusively in the subspecies *infantis*, provides a clue for an enlarged size of their genome compared to subspecies *longum* and *suis*. Nearly a half of these orthologous groups conserved in *B*. *longum* subsp. *infantis* comprised genes encoding hypothetical proteins with no functional assignment. However, the remaining part of subsp. *infantis*-specific orthologous groups could play an important part in cell metabolism and adaption to host GIT environment. A total of 33 groups were found to be involved in the transmembrane transport of various molecules, including phosphonates (CP001095, Blon_0021-Blon_0022), carbohydrates (Blon_2360-Blon_2361), and ferrous iron (Blon_1648). Also, the subsp. *infantis* 'core addition' orthologous groups include genes, encoding L-fucose mutarotase (Blon_2305), which represents a component of fucose utilization pathway, putative glyoxalase (Blon_1645), various glycoside hydrolases (mainly sialidases and fucosidases), signal transduction histidine kinases, and transcription regulators. The core additions for other subspecies were significantly smaller. Only 19 orthologous groups were specifically conserved in subsp. *longum* and absent from other subspecies. Functions of the proteins encoded by the most of them were unknown. Among the identified products were the spermidine synthase (CP000605, BLD_0231) and the carbon starvation protein CstA, probably involved in peptide uptake (BLD_0646). Core addition set for subsp. *suis* comprised only 12 groups, encoding putative beta-lactamase (JGZA01000015, BLSS_0128), transporters and hypothetical proteins. The lists of all ‘core additions’ are located in Supporting Information (S1, S2 and S3 Files).

### Pan-genome of *B*. *longum* subsp. *longum*

A choice of bacterial strains may significantly affect pan-genome analyses, regardless of the model used to interpret the data [45]. To date, the subspecies *longum* has been studied far more thoroughly than other subspecies of *B*. *longum*, and 23 out of 28 available genomes belong to this clade. While the representatives of this subspecies form the main part of our dataset, the members of subspecies *infantis* and *suis* represent outliers by their gene content. Such sampling heterogeneity may potentially lead to the biased estimations of pan-genome characteristics. Thus, for the analysis of pan-genome features we selected only the strains of *B*. *longum* subsp. *longum*, while the other two subspecies were not analyzed due to extremely low number of strains sequenced.

**Fig 5:**
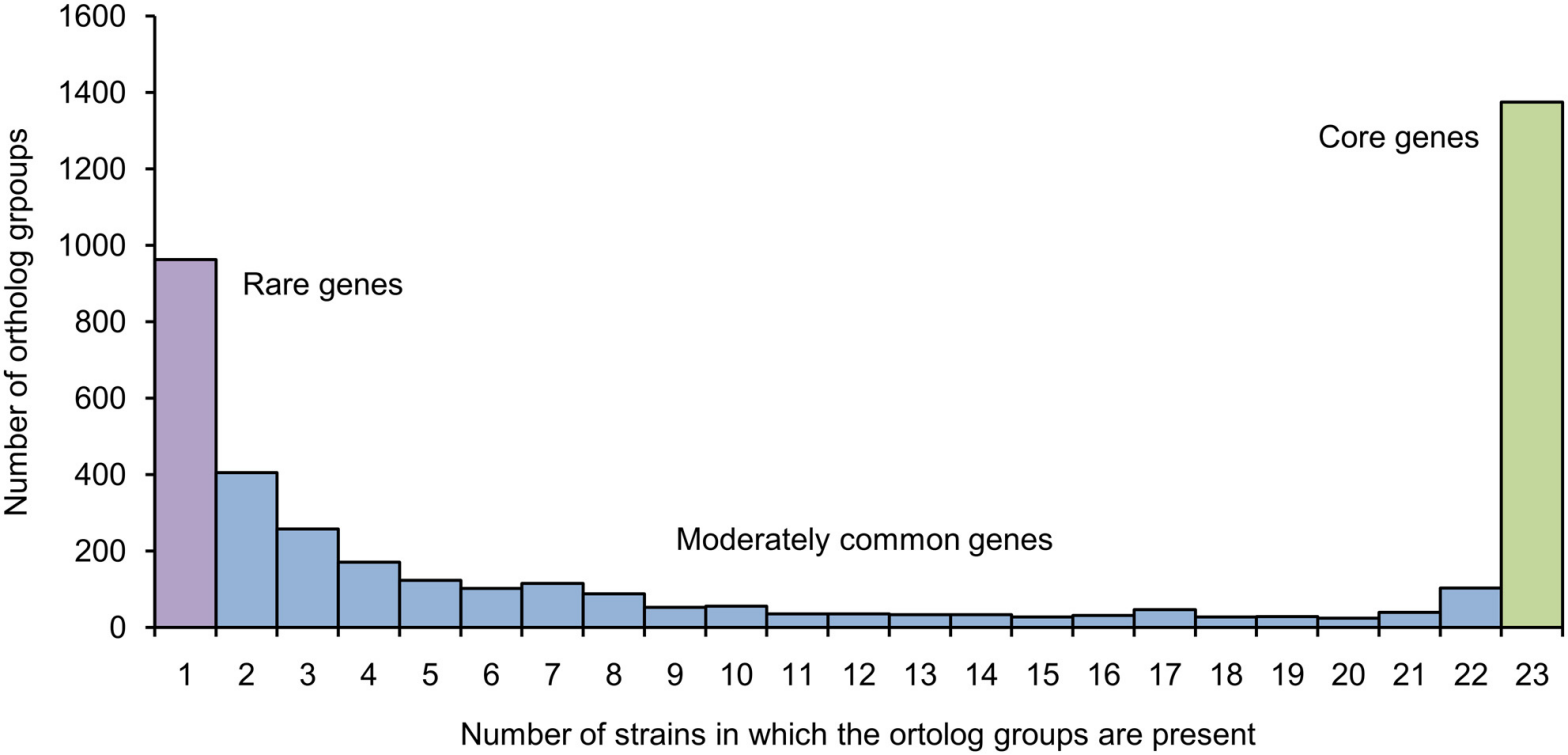
Distribution of ortholog groups by the number of strains in which they are present. Ortholog groups were divided into three categories based on their prevalence.

The size of pan-genome inferred from genome sequences of 23 *B*. *longum* subsp. *longum* strains was 4169 orthologous groups, what represents nearly 78% of the total number of orthologous groups obtained for all three subspecies of *B*. *longum* (5324 clusters). This value was more than twice higher than the average number of ortholog clusters in an average strain of *B*. *longum* subsp. *longum* (2027±126). The distribution of orthologous groups by the number of strains in which they could be found had a typical slightly asymmetric U-shape form implying that most of the genes being either extremely rare or by opposite ubiquitous (Fig 5).

**Fig 6:**
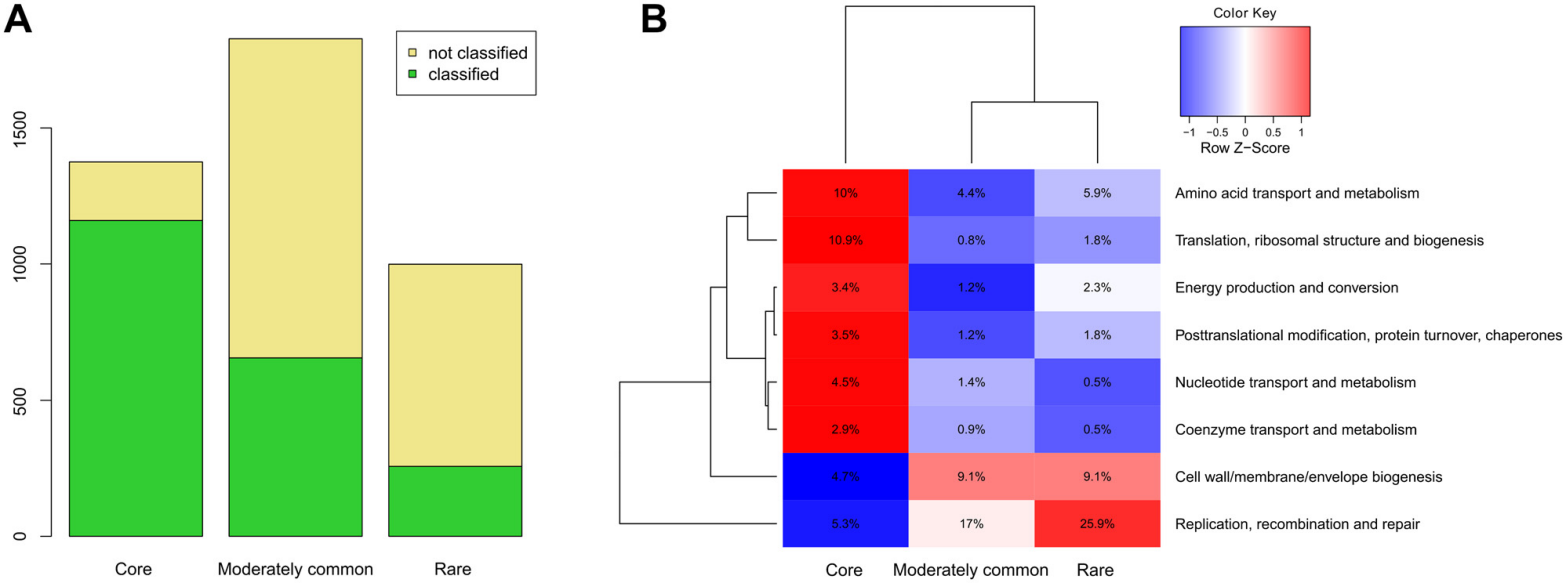
Functional annotation of ortholog groups in the different parts of pan-genome of *B*. *longum*. (A) Distribution of ortholog groups, functionally annotated and not annotated using search in the COG database in the different parts of the pan-genome. (B) Scaled heatmap of the distribution of the functional classes among the different parts of pan-genome. Only the classes with the non-uniform distribution among the parts are shown (Fisher’s exact test, Benjamini-Hochberg controlling procedure, q-value<0.05). The tree was inferred by complete linkage hierarchical clustering.

Based on this distribution we divided all orthologous groups in a pan-genome into three groups: core genes (present in all of the 23 *B*. *longum* subsp. *longum* strains), moderately common genes (present in several strains) and rare genes (seen in only one out of 23 strains). To determine the differences in functional annotation of clusters within these groups we performed re-annotation of all orthologous groups in a pan-genome using COG database (Fig 6), which provides imperfect but simple and concise functional classification. In total, we obtained 1160 clusters with predicted functions among the core genes, 660 clusters among the moderately common and 220 clusters among the rare genes. The rest of the 51% of orthologous groups could not be annotated using COG database and most of these genes were located among the moderately common genes and rare genes (Fig 6A). The functional annotation showed the non-uniform distribution of several functional classes among functionally annotated genes (Fig 6B). The core genome was found to be enriched with genes involved in translation and posttranslational modification, energy production and conversion, as well as genes for amino acid, coenzyme, and nucleotide transport and metabolism. On the contrary, the genes for cell envelope biogenesis were overrepresented among the moderately common and rare genes, but not the core genes, that reflecting the high level of diversity of various glycosyltransferases, carbohydrate modification proteins and sortases in the pan-genome of *B*. *longum* subsp. *longum*, defining the strain-specificity of surface molecules of these bacteria. The 'Replication, recombination and repair' group was overrepresented among the rare orthologous groups, referring to the genes of mobile elements, such as transposases and site-specific recombinases, distribution of which was found to be nearly strain-specific. Interestingly, carbohydrate transport and metabolism genes although representing a large part of the pan-genome were uniformly distributed among all three groups of COG-annotated genes (9.6% of core, 13.5% of moderately common and 9.6% of rare genes).

**Fig 7:**
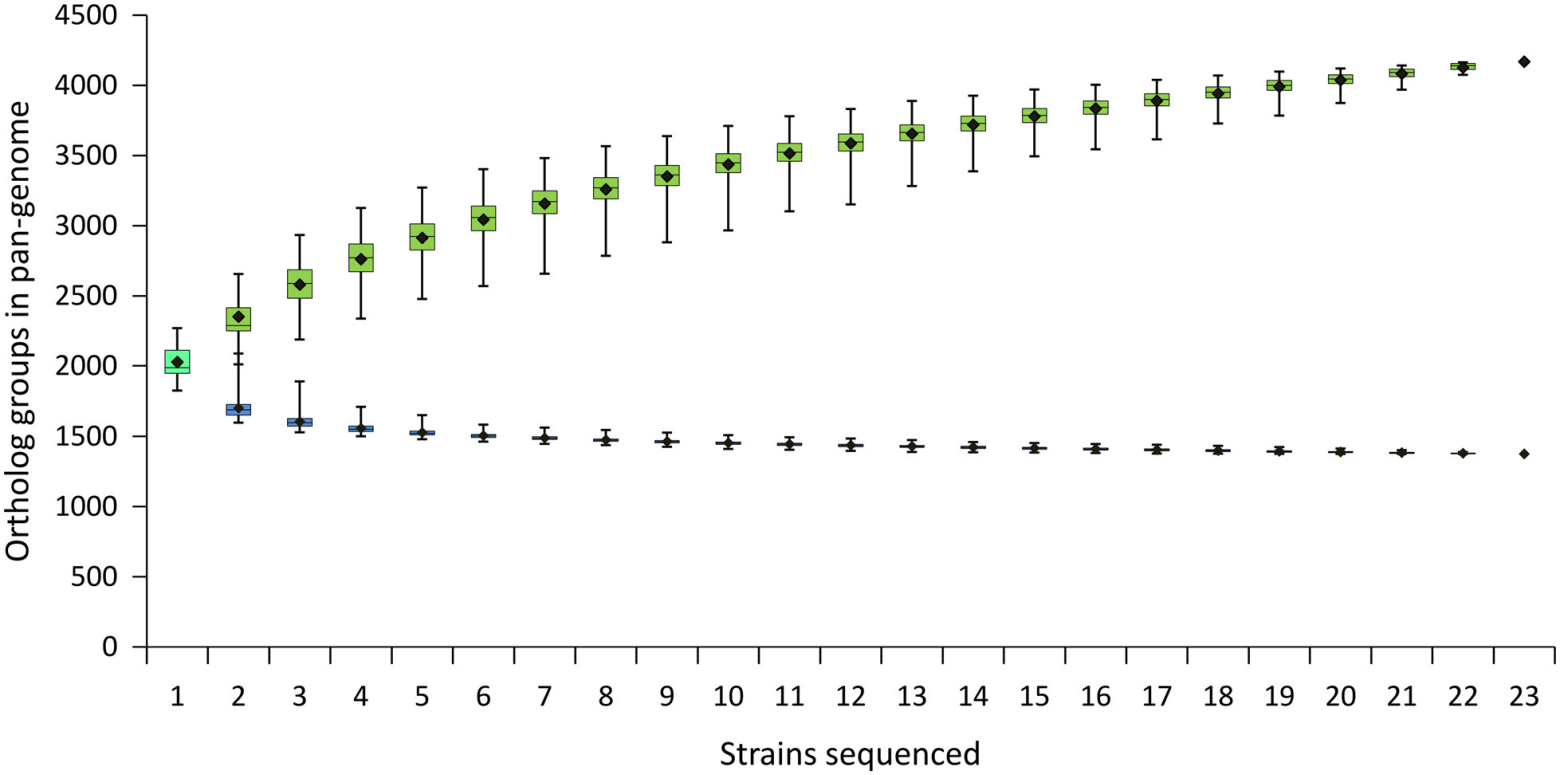
Sizes of pan-genome and core genome of *B*. *longum* subsp. *longum* as functions of the number of strains sequentially added. Each dot represents distribution of data obtained from 10000 random permutations of strain order. Central horizontal lines, medians; lower and upper border of boxes, 25 and 75 quartiles, respectively; ends of the whiskers, minimal and maximal values; black dots, mean values.

To estimate the sizes of the core and the pan-genome of *B*. *longum* subsp. *longum* the plot was built that reflects the number of genes present in the core- and the pan-genome as a function of the number of individual genomes (Fig 7). The absolute difference between mean and median sizes of pan-genome in each point was calculated to be 5.7±2.1 orthologous groups, showing the relative homogeneity of strain samples in comparison with the extreme cases described by Tettelin *et al*. [45]. Thereby, we used only the mean values for further analysis. The core genome size was found to be well approximated by a sum of power law and decreasing linear function (y = 571.8x^-1.167^–4.39x + 1459.1, R^2^ = 0.99998). With addition of the last 3 strains in the set the core genome lost on average 4–5 ortholog groups for each added strain. However, this small decline in the right portion of the curve could be a noise coming from draft genome sequences. Thus it cannot be excluded that the real core genome function reaches plateau. The mean values of the pan-genome size were found to be approximated (R^2^ = 0.999995) by power law equation y = 2007x^0.233^. Addition of the last 3 strains in the set increased the pan-genome for 42–45 ortholog groups on average for each added strain.

**Fig 8:**
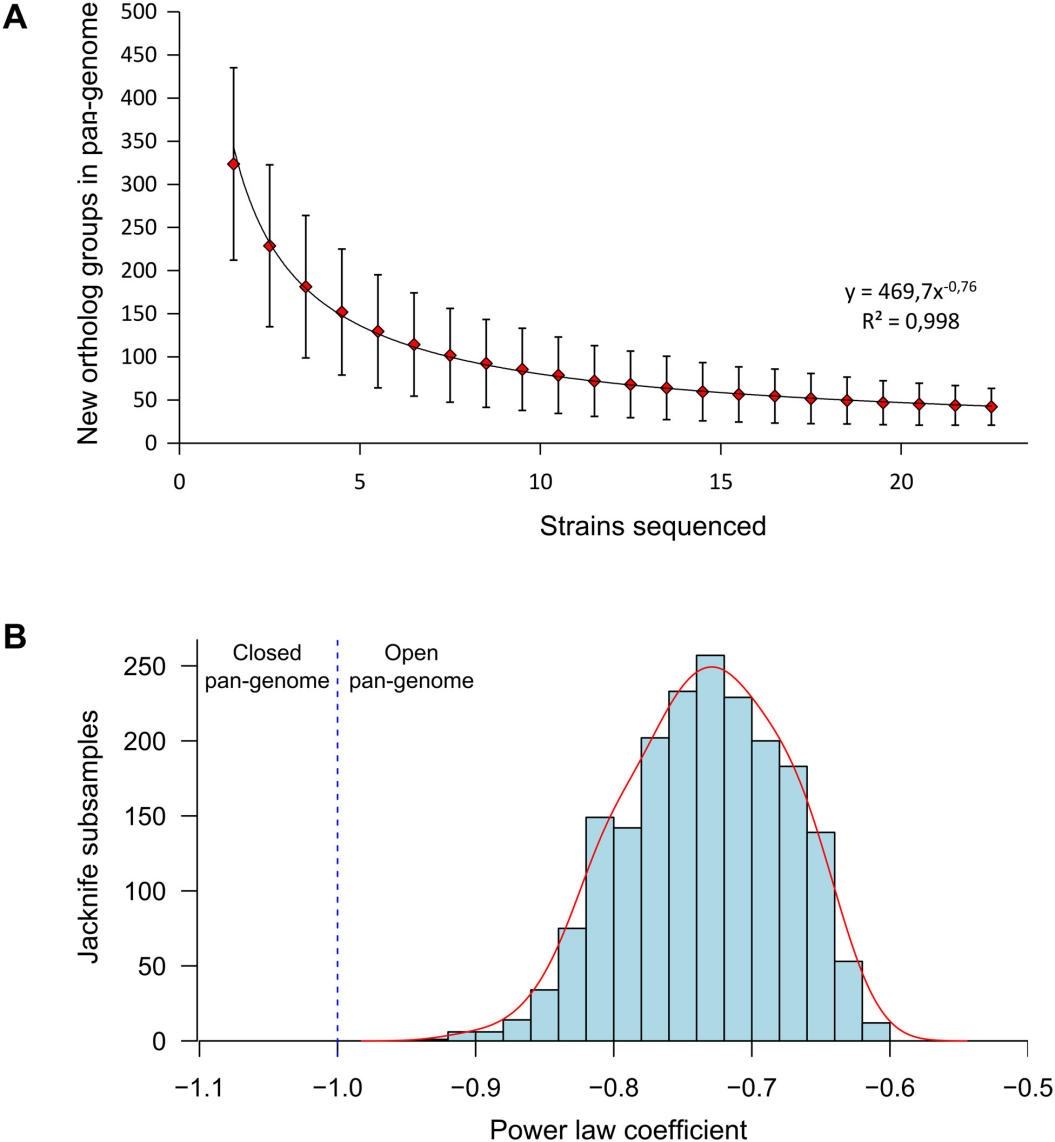
*B*. *longum* subsp. *longum* pan-genome according to the power law model. (A) Plot of number of new ortholog groups as a function of the number of strains sequentially added. Each dot represent mean value of data obtained from 10000 random permutations of strain order. Solid line show the power law n = kN^-α^ least squares fit, where N is the number of sequenced strains. (B) Distribution of power law coefficient (α) in jackknife procedure including 2000 iterations. Half of the strains were randomly discarded in each iteration, power law least squares fits were computed using mean values of data obtained from 1000 random permutations of strain order. Solid line shows the border between open pan-genome and closed pan-genome areas.

The number of new ortholog groups added to pan-genome with the inclusion of new genomes was approximated (R^2^ = 0.998) b equation y = 469.7x^-0.76^ (Fig 8A). The power law coefficient within this equation was located between -1 and 0, corresponding to the typical open pan-genome model [45]. Assuming that an open genome model is applicable to B. *longum* subsp. *longum* a very large number of sequenced genomes would be needed to characterize the entire gene repertoire of the subspecies. According to the function inferred, more than 3000 added strains would be required to lower the increase of pan-genome to 1 ortholog group for each added strain. The pan-genome of B. *longum* subsp. *longum* can be considered to be less ‘open’ than that of *Pantoea ananatis* or *Streptococcus agalactiae*, but more ‘open’ than that of *Streptococcus pneumonia* or *Listeria monocytogenes* [65–68].

Even within the subsp. *longum* that we used for our analysis of pan-genome characteristics the strain sampling was not fully homogenous, because closely related strains (44B and 1-6B, 2-2B and 35B and 7-1B) were present in our sample and could lead to an underestimation of genetic diversity. To estimate the influence of inhomogeneous strain sampling on the pan-genome analysis we performed half-size subsampling procedure. The power law coefficient of the increase of pan-genome size varied from -0.60 to -0.92 in the subsamples with the mean value of -0.73. No values indicating a closed pan-genome structure were obtained (Fig 8B).

The other possible sources of bias could be too stringent or too soft criteria of clustering of protein-coding genes into ortholog groups. OrthoMCL procedure begins with all-against-all blastp comparison of a full set of proteins from studied genomes, using relatively low thresholds (50% sequence identity, e-value 1e-5). On the next step the obtained similarity matrix is weighed and protein sequences are clustered using MCL algorithm. The tightness (granularity) of clusters is determined primarily by the inflation index, I. For this study we used I = 1.5 as recommended by Li *et*. *al*. [44]. To ensure the open structure of *B*. *longum* subsp, *longum* pan-genome we also used values 1.1 and 4.0 as described in [44]. With these parameters the pan-genome of *B*. *longum* subsp. *longum* had a size of 3877 and 4350, respectively. The power law coefficient calculated for I = 1.1 and I = 4.0 was -0.74 and -0.79, respectively, which in either way corresponds to an open pan-genome. Conversely speaking, even by changing the granularity of clustering over a broad range of values we did not obtain results which contradicts to the open pan-genome structure.

Another obstacle towards understanding a pan-genome structure of *B*. *longum* susbp. *longum* population is the large number of ORFs coding for hypothetical proteins. The average genome of *B*. *longum* subsp. *longum* was found to contain 572 orthologous groups (28% of the total number of groups) not classified using COG database. The majority of these groups (79%) lack any conservative domains that belong to Pfam-A or Pfam-B categories of families [47]. In the pan-genome of *B*. *longum* subsp. *longum* non-classified groups accounted for 51% of the total number of orthologous groups (see S6 and S7 Figs for comparison with other subspecies). The large number (50.0%) of short ORFs (<300 bp) among the non-classified genes suggests that many orthologous groups lacking functional annotaions could actually correspond to the mis-annotated ORFs (S8 Fig). Another major fraction of the non-classified orthologous groups could be represented by rare genes with obscure or unknown functions; these genes are mainly found within the putative prophage sequences of *B*. *longum* subsp. *longum*. To estimate the impact of these ORFs with unknown or possibly absent functions on the pan-genome equation we expressed the number of new orthologous groups as a function of the number of strains with all non-classified orthologous groups excluded, and obtained the equation y = 168,0x^-0,92^ (R² = 0,999). The power law coefficient in this equation implies that the pan-genome remains open. Moreover, with the subsequent exclusion of 'Replication, recombination and repair' category orthologous groups, which are enriched by genes of selfish genetic elements, we obtained the equation y = 141,9x^-0,97^ (R² = 0,999); showing that the pan-genome in this case is still open and the number of orthologous groups follows a nearly logarithmic trend with addition of new strains [45]. Therefore, the open pan-genome of *B*. *longum* subsp. *longum* couldn't be ruled out even with the use of the strictest approaches, allowing us to conclude, that by addition of a large number of new genomes of *B*. *longum* subsp. *longum* we will continue to discover new genes, reflecting the vast genetic and phenotypic diversity of this species.

### Glycoside hydrolase genes in *B*. *longum*

It is generally recognized, that the metabolism of bifidobacteria is specialized in the fermentation of a wide spectrum of complex carbohydrates. Several studies have discerned the enzymes and pathways for degradation of plant-derived, animal milk-derived, and human milk glycans [69–71]. However, our analysis of *B*. *longum* genomes available to date shows that although the indispensable final stages of carbohydrate metabolism (galacto-N-biose/lacto-N-biose pathway and fructose-6-phospate phosphoketolase pathway) are encoded in all studied genomes, the spectrum of glycoside hydrolases responsible for the utilization of complex carbohydrates, shows significant inter-strain diversity.

**Fig 9:**
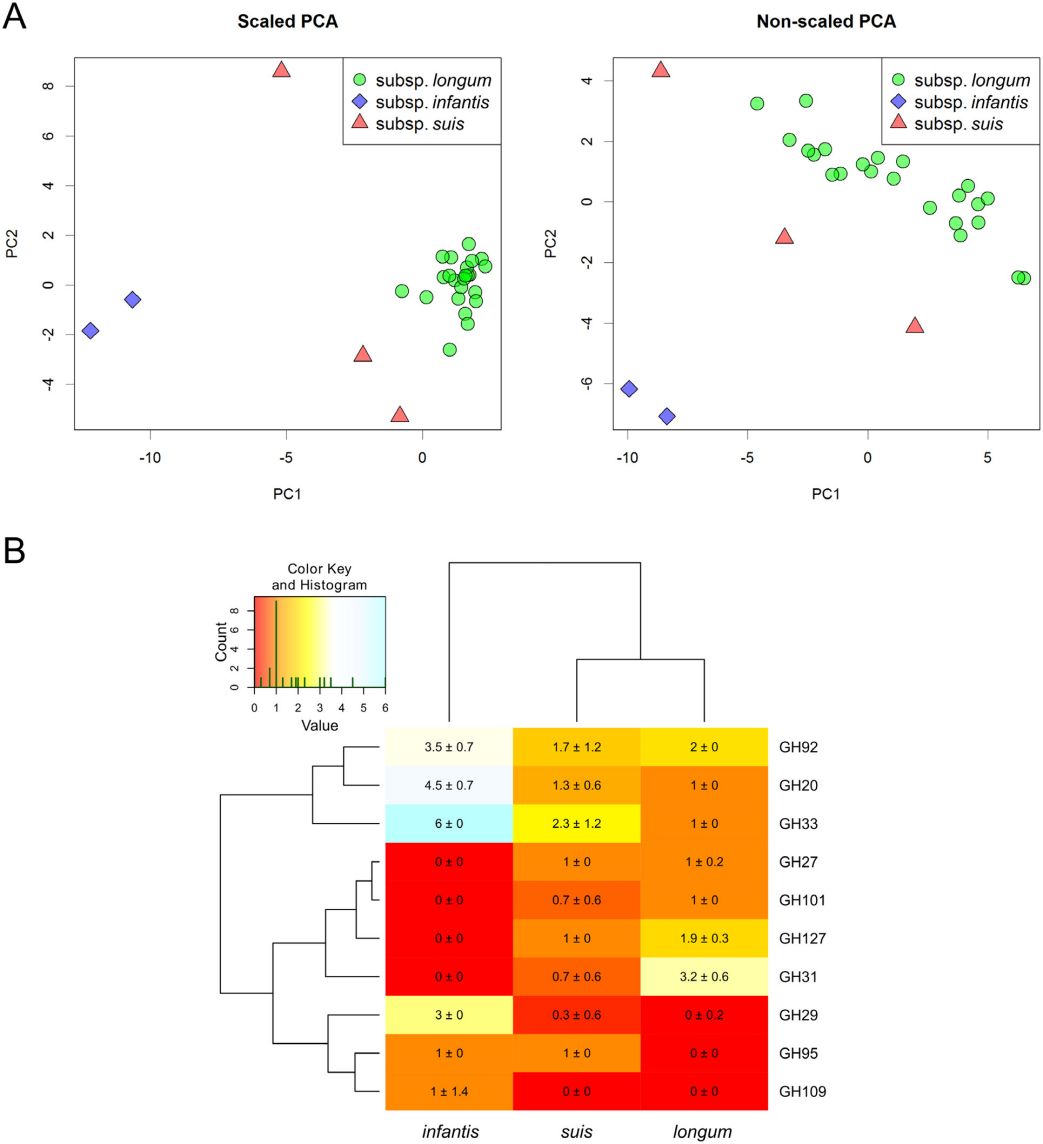
Glycoside hydrolase genes in genome sequences of *B*. *longum*. (A) Principal component analysis of the number of members of glycoside hydrolase families in genome sequences of *B*. *longum*. Subspecies *longum*, green circles; subspecies *infantis*, blue diamonds; subspecies.*suis*, red triangles. (B) Heatmap, representing the number of members of glycoside hydrolase families in *B*. *longum* strains. Only the families with non-uniform distribution are shown. The tree was inferred by complete linkage hierarchical clustering.

Using CAZymes Analysis Toolkit we annotated glycoside hydrolase genes, and then excluded the families of enzymes targeting peptidoglycan, leaving only the enzymes involved in carbohydrate catabolism. The numbers of genes within each glycoside hydrolase family were used for principal component analysis. We employed either, a scaled representation of data, allowing minor families to have significant impact on the result, or a non-scaled representation that maximizes the impact of the abundant families. The subspecies of *B*. *longum* were found to be clearly distinct using both approaches. The genomes of *B*. *longum* subsp. *longum* and *B*. *longum* subsp. *infantis* were found to be quite distant from each other, while the strains of *B*. *longum* subsp. *suis* formed an intermediate 'layer' between the former two (Fig 9A).

Ten glycoside hydrolase families were found to be non-uniformly distributed between the subspecies (Fig 9B). These genes can be grouped into three clusters. The first one contains families that are abundant in the genomes of *B*. *longum* subsp. *infantis* and scarcely distributed in other subspecies. This cluster contains family GH92, comprising exo-acting α-mannosidases, family GH20, including exo-acting N-β-acetylglucosaminidases and exo-acting lacto N-biosidases, and the family GH33 that contains sialidases. The second cluster contains the genes that are absent from *B*. *longum* subsp. *infantis*, but could be present in either *B*. *longum* subsp. *longum* and *B*. *longum* subsp. *suis*. It includes family GH27 that combines various enzymes, including α-galactosidase and β-L-arabinopyranosidase; family GH101, comprising endo-α-N-acetylgalactosaminidases; family GH127, containing β-L-arabinofuanosidases, and family GH31, comprising a diverse group of enzymes with α-glucosidase and α-xylosidase activities. The third cluster combines families that seem to be nearly absent from *B*. *longum* subsp. *longum*. This cluster contains families GH29 and GH95, comprising α-L-fucosidases, and family GH109, including α-N-acetylglucosaminidases, operating via NAD-dependent hydrolysis mechanism.

In agreement with the previous studies [6,18] genomic sequences of *B*. *longum* subsp. *longum* and *B*. *longum* subsp. *infantis* reflect the specialization in the catabolism of different types of carbohydrate nutrients. *B*. *longum* subsp. *longum* exhibits higher genomic capacity to utilize the plant-derived glycans, including arabinoxylans, while *B*. *longum* subsp. *infantis* is more suited for the degradation of human milk oligosaccharides that contain characteristic residues of fucose and sialic acid. Interestingly, *B*. *longum* subsp. *suis* appears to have an 'intermediate' carbohydrate catabolism capacity lying between *B*. *longum subsp*. *infantis* and *B*. *longum* subsp. *longum*. No families of glycoside hydrolases were significantly overrepresented or underrepresented in *B*. *longum* subsp. *suis* compared to the other two subspecies.

### Plasmids in the sequenced strains of *B*. *longum*

The small cryptic plasmids, belonging to multiple families and having different modes of replication are known to be widespread in *B*. *longum* subsp. *longum* [6]. For example, strain DJO10A harbors two plasmids, the larger one (pDOJH10L) was shown to be a cointegrate, containing regions of high similarity with the well-studied plasmids pNAC2 and pKJ50, while the smaller one (pDOJH10S) have no known homologs [72]. The strain 157F was also found to harbor two plasmids, one of which (p157F-NC1) was similar to pDOJH10L, and the other one belonged to a widespread family, including the plasmid pKJ36 (p157F-NC2) [56]. The plasmids found in the strain KACC 91563 include a large plasmid with a large region of homology to a pB80-like plasmids (BLINAS_P1) and a cointegrate molecule composed of two members of pKJ36 family (BLINAS_P2) [25]. These examples show not only the diversity of *B*. *longum* subsp. *longum* plasmids, but also the combinatorial nature of their variability. However, no plasmids have been discovered in the other two subspecies of *B*. *longum*.

**Fig 10:**
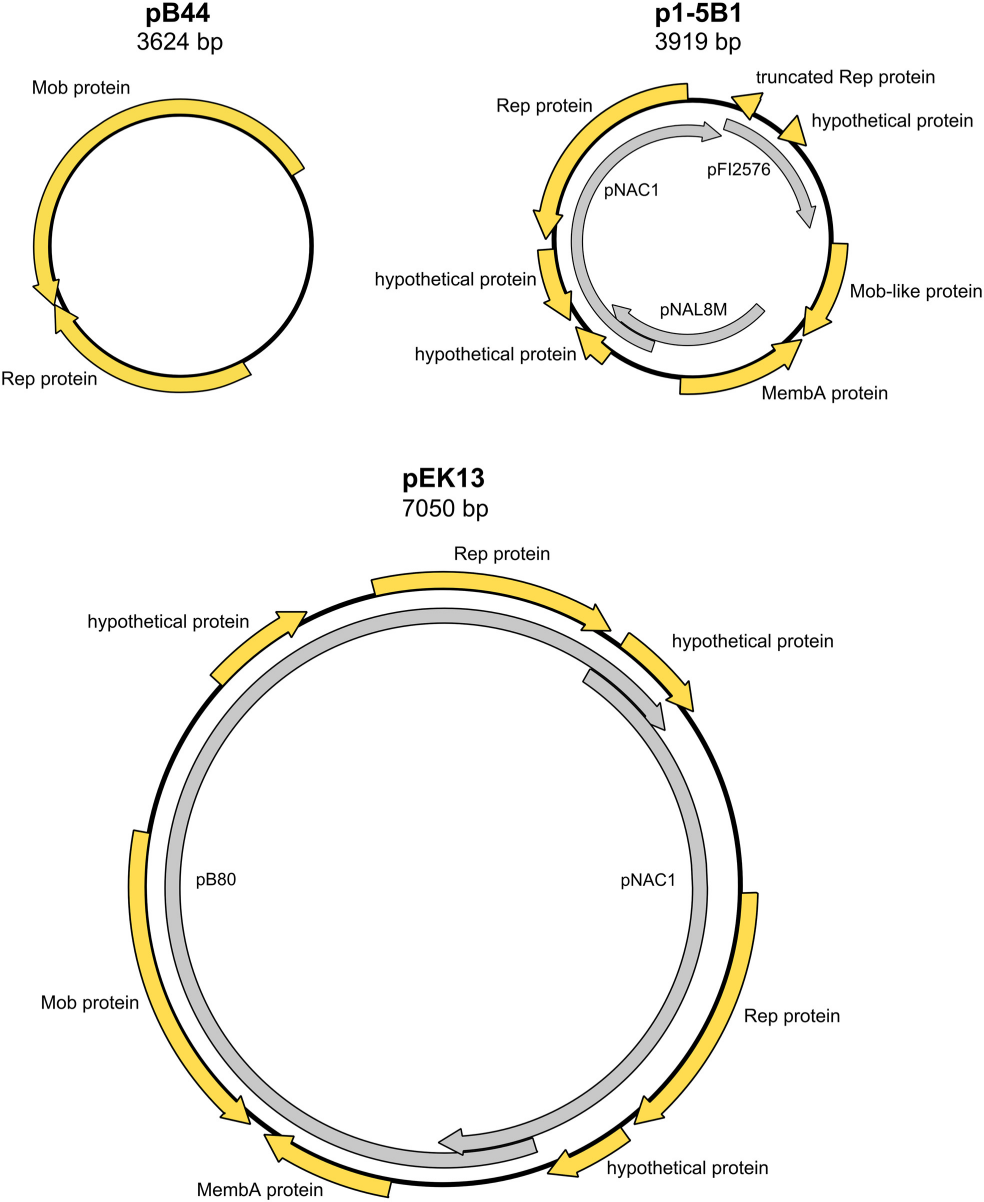
Families of small cryptic plasmids, discovered in the strains, sequenced for this study. Outer yellow arrows, putative open reading frames; inner grey arrows, fragments homologous to other known plasmids.

In this study we surveyed the genomic sequences of *B*. *longum*, sequenced for this study, for the presence of small cryptic plasmids. In total, 8 out of 12 strains were found to contain putative plasmid sequences (Table 3). All of them were circularizable based on the repeated sequences at the ends of contigs extended by read mapping (Fig 10). The plasmid-to-genome coverage ratio varied between 4.0 and 20.2 for all plasmids studied, with the exception of putative chromosome-integrated plasmid from strain 35B, represented by a separate contig with plasmid-to-genome coverage ratio 1.0.

**Table 3:** Small cryptic plasmids from *B*. *longum* strains sequenced for this study.

Plasmid	Strain	Plasmid-to-chromosome coverage ratio	Length of presumptive circular sequence, bp	Accession number
p1-5B1	1-5B	10.5	3919	KP691640.1
p1-6B1	1-6B	4.9	3919	KP691636.1
p17-1B	17-1B	13.2	3919	KP691641.1
p1-5B2	1-5B	6.6	3624	KP691639.1
p1-6B2	1-6B	4.0	3624	KP691635.1
p35B	35B	1.0	3624	AJTI01000126.1
p2-2B	2-2B	5.4	3624	KP691633.1
pB44	VMKB44	20.2	3624	AY066026.1
p72B	72B	11.0	3624	KP691638.1
pEK13	EK13	12.7	7050	KP691637.1

Most plasmids discovered had a length of 3624 bp and belonged to the well-studied family of bifidobacterial plasmids, which includes plasmid pKJ36 and pB44 [52]. Members of this family are known to be widely distributed among *B*. *longum* subsp. *longum* [6]. Additionally, the plasmid pBBKW-1, isolated from *B*. *kashiwanohense*, seems to be a cointegrate containing two copies of a pKJ36/pB44 family plasmid. This family of plasmids replicates through the rolling circle mechanism and carry genes coding for replication protein (Rep), mobilization protein (Mob), and other proteins of unknown function, such as the transmembrane protein MembB. Plasmids of this family have been used for the construction of shuttle cloning vectors, including one of the plasmid from this study, pB44 of *B*. *longum* subsp. *longum* VMKB44 [52]. Sequence comparison has shown, that pKJ36/pB44 family plasmids p1-5B2 and p1-6B2 found in strains 1-5B and 1-6B (both isolated from Child 1 at the age of 5), respectively, shared 100% identity with each other and 99.5% identity with pB44. Plasmids p35B and p2-2B found in strains 35B and 2-2B (isolated from Child 2 in two different time points), respectively, were also completely identical to each other, almost identical to p72B (99.9% identity), and closely related to the p1-5B2/p1-6B2 pair of molecules (98.8% identity, see S9 Fig for a phylogenetic tree of pKJ36/pB44 family plasmids).

Other putative small cryptic plasmids found in genomic sequences didn't have the whole length homology to the previously described plasmid families, but seem to originate from other plasmids through a series of recombination events.

Plasmids p1-5B1, p1-6B1, and p17-1B found in the strains 1-5B, 1-6B, and 17-1B, respectively, had 100% identical sequences of 3919 bp. These molecules are comprised of three blocks of sequence highly homologous to the previously described bifidobacterial plasmids pNAL8M (1068 bp, 99% identity), pNAC1 (1957 bp, 89% identity) and pFI2576 (719 bp, 92% identity) [73–75]. The ORFs mapped on these plasmids include genes for Rep protein homologous to pNAC1 replication protein and MembA protein homologous to that encoded by pNAL8M. A fragment of the *rep* gene homologous to pFI2576 Rep, and an ORF coding for a hypothetical protein with 30–50% identity to known Mob proteins of bifidobacteria were also mapped on plasmids from this group. Products of the other reading frames of this plasmid were hypothetical proteins with no function assigned.

The plasmid pEK13 from the strain EK13 has a length of 7050 bp, and is larger than most known cryptic plasmids in bifidobacteria. The nucleotide sequence includes a fragment corresponding to nearly the whole length of a previously described plasmid pB80 [52] of *B*. *bifidum* (4978 bp, 99% identity). Another large fragment of pEK13 is homologous to the plasmid pNAC1 (2885 bp, 96% identity). The two regions of homology overlap each other at both ends giving rise to the two imperfect repeats of 404 and 408 bp, which share 82.5% identity. Moreover, in each of these overlaps one half is more similar to one of the 'parental' plasmids, and the other half is more similar to the other 'parental' plasmid. This structure unambiguously indicates that the plasmid pEK13 originated as a result of cointegration of two ancestral plasmids through homologous recombination. Interestingly, a 1 kb region of the plasmid pNAC1 that is absent from pEK13 is located upstream of the *rep* gene and is flanked by the origin of replication and putative recombination site. One can speculate that its loss was a separate event before or after the co-integration event. The resulting sequence of pEK13 includes genes for two Rep proteins (both seem to be functional), Mob protein, MembA protein, and several hypothetical proteins.

A recent study has shown the existence of conjugative megaplasmids among several members of the genus *Bifidobacterium*, including *B*. *longum* subsp. *longum* strains 44B, 1-6B, and 2-2B [59]. A characteristic feature of these plasmids is the presence of an unusual CRISPR-Cas system (discussed below).

### CRISPR-Cas and restriction-modification systems in *B*. *longum*

CRISPR-Cas systems provide prokaryotes with the mechanism of adaptive heritable resistance against phages and plasmids mediated by the incorporation of foreign DNA fragments into prokaryotic genome with subsequent cleavage of corresponding foreign DNA or, in some cases, RNA molecules [76]. These systems are widely distributed in prokaryotes, and in many cases the distribution seems to be strain-specific. Due to the hypervariable nature of CRISPR loci, the study of their sequence may allow for the investigation of a strain history with higher resolution and accuracy compared to other methods.

**Fig 11:**
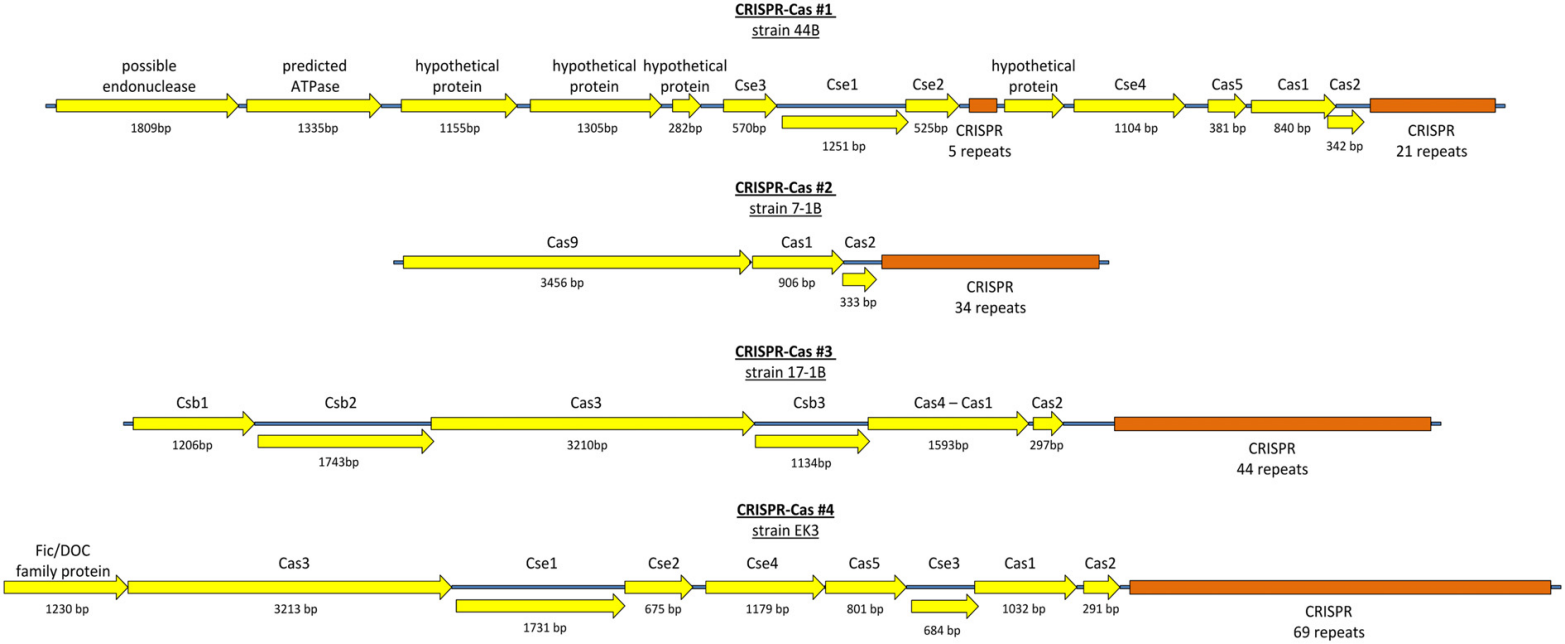
Structure of CRISPR-Cas systems in *B*. *longum*. Each scheme represents a typical locus from one of the studied strains.

In our analysis of genomic sequences of *B*. *longum* we discerned 4 distinct CRISPR-Cas system types (Table 4, Fig 11, S10 Fig). The first type (#1) has recently been shown to be a part of a megaplasmid in strains 44B, 1-6B and 2-2B [28,59]. This system has new and a particularly unusual structure: it includes one set of *cas* genes and two CRISPR loci with repeat sequences that differ in one nucleotide. This system has been described by Bottacini *et al*. [59] as two different adjacent systems, but the single set of *cas* genes (including essential genes *cas1* and *cas2*, each present in a single copy) contradicts the idea of independence of these two systems. Thus we suppose that considering this region as single system may better reflect its properties. The rule "spacers closest to *cas* genes are variable and the distant ones are conservative" [77] works for both of the CRISPR loci in this system. The conservative spacers are located at the 3' end of the loci (by the direction of *cas* genes), showing that the both loci can acquire new fragments of foreign DNA. A region of such structure could originate by translocation of a fragment of CRISPR locus or by fusion of two homologous but diverged adjacently located systems. System's #1 Cas locus contains genes, coding for the proteins of Cascade complex, which is typical for bacterial CRISPR-Cas class I systems [76]. However, it lacks the *cas3* gene that is indispensable in the prototype systems of this class. Interestingly, strains 44B and 1-6B isolated from child 1 had exactly the same spacers sequences, reflecting the common origin of these two strains. However, despite the common origin of strains 2-2B, 35B, and 7-1B, the CRISPR-Cas system #1 was only present in strain 2-2B and was absent in the genomic sequences of strains 35B and 7-1B, which was additionally confirmed by PCR.

**Table 4:** CRISPR-Cas systems found in the *B*. *longum* genome sequences.

System number	System type	Repeat sequence	Strains, where the system is present
1	N/A	ATCTACCCCGCACATGCGGGGATAAACC (locus 1), ACCTACCCCGCACACGCGGGGATAAACC(locus 2)	44B, 1-6B, 35B
2	Class II	CAAGCTTATCAAGAAGGGTGAATGCTAATTCCCAGC	44B, 35B, 7-1B, DJO10A, 12_1_47BFAA, KACC 91563, CECT 7347
3	dpsyc variant	CTTGCATACGTCAAAACGTATGCACTTCATTGAGGA	17-1B
4	Class I	GTTTGCCCCGCATGCGCGGGGATGATCCG (BBMN68), GTTTGCCCCGCACGCGCGGGGATGATCCG (EK3)	BBMN68, EK3

Other CRISPR-Cas systems in *B*. *longum* belong to the known types, and their *cas* proteins have multiple homologs in other species of bifidobacteria as well as in the other members of the phylum *Actinobacteria*. The system #2, which is related to the well-studied CRISPR-Cas class II, is widely distributed among the strains of *B*. *longum* and present in strains 44B, 35B, 7-1B, DJO10A, 12_1_47BFAA, KACC 91563, and CECT 7347. The CRISPR loci of the strains 2-2B, 35B, and 7-1B, isolated from child 2, are identical with the exception of one spacer (third from the 3’-end) that was absent from the strain 2-2B. The strains 44B and 1-6B, isolated from Child 1, have the same spacers in the sequenced regions of CRISPR loci of this system, however, we did not succeed in obtaining a full-length PCR product from strain 1-6B, suggesting that the integrity of the locus is disrupted in the genome of this strain. This system is also possibly damaged by translocation of the 3'-end of the locus (if not a result of mis-assembly) in the strain 12_1_47BFAA and by the insertion of IS256 family IS element in the leader sequence of the CRISPR locus in the strain KACC 91563.

The system #3 belongs to the *Dpsyc* type of CRISPR-Cas systems. In *B*. *longum* it could only be found in strain 17-1B. The system #4 has a typical structure of the CRISPR-Cas class I. The two similar #4 systems were found in strains BBMN68 and EK3, differing by only one nucleotide in their repeat sequences. Also, the CRISPR locus in BBMN68 is damaged by the insertions of IS elements, belonging to the families IS21 and IS3.

Another major contributors to the bacterial defense against plasmids and bacteriophages are the restriction-modification (R-M) systems. These systems generally act by methylating specific sites in bacterial DNA and digesting and eliminating DNA molecules, containing non-modified sites. Using the REBASE database [36] search with subsequent manual curation we detected 12 distinct orthologous groups of putative restriction endonuclease genes in the genomes of *B*. *longum* strains, eight of which were located adjacent to the predicted DNA methylase genes (and the predicted specificity proteins genes in case of type I systems). No restriction endonucleases genes were found to be present in the core genome of the species. The most widespread restriction modification systems were: one type I system (BLD_1959—BLD_1962 in the strain DJO10A) often clustered with the Mrr type IV restriction endonuclease (BLD_1958) and *Eco*RII-like type II system (BLD_0355—BLD_0356 in the strain DJO10A). These two systems were present in 20 and 18 strains, respectively. Several strains were also found to contain a predicted type III restriction modification system (BL171B_01860—BL171B_01865 in the strain 17-1B). The total number of restriction endonucleases per genome in *B*. *longum* varied between 1 and 6 (median of 3). The genomes of *B*. *longum* subsp. *infantis* possessed only one putative restriction endonuclease gene (BLIJ_0294 in the strain ATCC 15697), which contains a frameshift in the strain ATCC 15697, but seems to be intact in the strain EK3. The encoded protein shares 79% identity with the restriction endonuclease BbrUIIIR from *B*. *breve* (Bbr_1118 in strain UCC2003). However, the adjacent regions lack any detectable DNA methylase genes *B*. *longum* subsp. *infantis* strains, suggesting that this endonuclease gene is either inactive or has changed its function.

### Genes potentially affecting bacterial competition and colonization

An important task in the genomics of bifidobacteria is to establish the genes responsible for the major components of probiotic activities, such as the ability to colonize the intestinal ecological niche, the immunomodulatory effect, and the ability to interfere with pathogenic microbes. Undoubtedly, the study of abundance and diversity of these genes could also be extremely important for the understanding of the forces shaping composition of the intestinal microbiota in general, as well as for the targeted search for candidates for probiotic strains and for genetic engineering-based improvement of probiotic strains. In our study we attempted to find in the sequenced genomes of *B*. *longum* strains some of the known genes whose involvement in probiotic activities of *B*. *longum* was confirmed experimentally. The results of the search are represented in Table 5.

**Table 5:** The presence of genes, involved in intestinal colonization and microbial antagonism, in the sequenced strains of *B*. *longum*.

Locus name	Locus example	subsp. infantis		subsp. suis			subsp. longum																						
		ATCC 15697	EK3	JDM301	AGR2137	LGM 21814	157F	E18	JCM 1217T	BBMN68	NCC2705	KACC 91563	12_1_47BFAA	DJO10A	CECT 7347	F8	7-1B	35B	2-2B	1-6B	44B	ATCC 55813	1-5B	17-1B	CCUG 52486	EK5	EK13	72B	VMKB44
Type IVb tight adherence (Tad) pilus-encoding gene cluster	BLD_1295- BLD_1298 (DJO10A)	+	+	+	+	+	+	+	+	+	+	+	+	+	+	+	+	+	+	+	+	+	+	+	+	+	+	+	+
Prepilin peptidase TadV gene	BLD_0613 (DJO10A)	+	+	+	+	+	+	+	+	+	+	+	+	+	+	+	+	+	+	+	+	+	+	+	+	+	+	+	+
Putative transporter and adhesin gene	BLD_1516 (DJO10A)	+	+	+	+	+	+	+	+	+	+	+	+	+	+	+	+	+	+	+	+	+	+	+	+	+	+	+	+
Sortase-dependent pilus-encoding gene cluster	BLD_1467 (DJO10A)					+	+		+	+	+	+		+	+	+	+	+	+	+	+	+			+	+	+		+
Lantibiotic prepeptide gene	BLD_1648, BLD_1650 (DJO10A)	+											+	+	+														
Lantidiotic immunity protein	BLD_1652 (DJO10A)	+	+	+	+							+	+	+	+														
Gene of fructose transporter protecting from Shiga-like toxin	BLD_0045 (DJO10A)			+	+	+	+	+	+	+	+	+	+	+	+	+	+	+	+	+	+	+	+	+	+	+	+	+	+
Serpin gene	BLD_0126 (DJO10A)	+	+	+	+	+	+	+	+	+	+	+	+	+	+	+	+	+	+	+	+	+	+	+	+	+	+	+	+
Bile salt hydrolase gene	BLD_0536 (DJO10A)	+	+	+	+	+	+	+	+	+	+	+	+	+	+	+	+	+	+	+	+	+	+	+	+	+	+	+	+

The type IVb tight adherence (Tad) pilus-encoding gene cluster and the gene coding for prepilin peptidase TadV [38] were shown to be present in all strains of *B*. *longum*. Similarly, the putative ABC transporter shown previously to play a role in the adhesion [78] was also detected in all genomes. In contrast, the gene cluster encoding sortase-dependent pili [79], was found only in two thirds of *B*. *longum* strains.

Synthesis of lanthionine-containing bacteriocins (lantibiotics), known to possess a wide spectrum activity [80], may play an important role in the antagonistic activity of bifidobacteria against various pathogens. Clusters of lantibiotic biosynthesis genes were previously described in *B*. *longum* DJO10A and ATCC 15697^T^ [6,80]. In our study we also revealed their presence in several other strains (e.g. 12_1_47BFAA and CECT 7347). The structure of the gene clusters varies greatly between strains, suggesting that not all strains are actually capable of synthesizing a functional bacteriocin. However, the presence of defective gene clusters could be important for providing resistance to the corresponding lantibiotic.

Secreted serine protease inhibitor (serpin), which acts as an efficient inhibitor of pancreatic and neutrophil elastase [81], is not widespread in the genus *Bifidobacterium* [82]. Nevertheless, in our study all sequenced *B*. *longum* genomes contained the corresponding gene. The gene encoding bile salt hydrolase [83] also seems to be a part of the core genome of the species. The fructose transporter gene whose presence was shown previously to provide protection in an animal model of *E*. *coli* O157:H7 infection [56] was found in all genomes of *B*. *longum* except for the two strains of *B*. *longum* subsp. *infantis*.

### Comparison of the strains, isolated from the same patients

Several strains in this study were isolated from the same children at different ages. Comparison of these genomes could provide invaluable information about the strain dynamics in the intestinal microbiota during the long time periods and the evolutionary forces shaping the composition of intestinal microbiota in humans.

As stated above, the similarity within the sets of *B*. *longum* subsp. *longum* strains 44B —1-6B (isolated from child 1) and 35B —2-2B —7-1B (isolated from child 2) turned to be extremely high. The close relatedness can be inferred from nucleotide sequences of core genes, the composition of non-core genes, the sequences of such variable loci as CRISPR and the total average nucleotide identity. However, none of these strains were truly identical using each of these criteria, suggesting that all of these strains are genetically different even considering the draft status of their genomic sequences. Strains 2-2B and 35B could be the very close relatives other due to low amount of differences in housekeeping gene sequences. and the observed variations in gene content could be explained by a presence of megaplasmid [59] in 2-2B. The absence of 1 spacer in CRISPR-Cas #2 of strain 2-2B and the putative integration of plasmid in strain 35B are also minor differences. However, the strain *B*. *longum* subsp. *longum* 72B, isolated later from the same child, represents farther relative. Thus, the strains 35B, 2-2B, and 7-1B don't seem to represent any sort of 'lineage' by their relative positions on the phylogenetic tree, instead representing a random sample of a closely-related group of strains probably coexisting within the intestinal microbiome.

Strains 1-5B and 1-6B, isolated from Child 1 at the same time point, were found to be phylogenetically unrelated, but each had two different plasmids, both showing 100% identity between two strains. The plasmid p17-1B, identical to p1-5B1 and p1-6B1, has also been discovered in the different non-related strain 17-1B, isolated from the same patient five years later. This result could be explained by the high frequency of transmission of small cryptic plasmids between bifidobacteria. However, the mechanisms of such transmission have to be further elucidated. Taking into account the recent discovery of conjugative megaplasmids in bifidobacteria [59] one could speculate that small mobilizable plasmids use the Tra machinery of megaplasmids for mobilization with their own Mob proteins used as an interface for recognition of *cis*-located *oriT* sites [84].

## Conclusions

The taxonomy and systematics of *B*. *longum* species was subjected to multiple changes in the recent decades. The groups "*longum*", "*infantis*" and "*suis*" had been first described as three distinct species, followed by their unification as the biovars of *B*. *longum*. Finally the three groups were reclassified as three subspecies [17,85]. In this study, the members of the subspecies were found to be clearly distinct using nearly all genetic criteria—the concatenated sequences of multiple core genes, average nucleotide identity and the total gene content. Using the comparative genomics approach we also confirmed the different ecological specialization of the subspecies: overrepresentation of the genes involved in metabolism of the milk-derived oligosaccharides in subsp. *infantis* and the metabolism of the plant-derived carbohydrates in subsp. *longum*. However, this study (as well as the study [18]) revealed many cases of misidentifications between three subspecies, reflecting their general phenotypic similarity and elusiveness of the border between them, which complicates the classical methods of bacterial identification. While the sequencing of 16S rRNA is known to be a robust method for discriminating between human-associated subspecies *longum* and *infantis* [29], the subspecies *suis* may be easily misidentified.

According to the data obtained using ClonalFrame, the overall impact of HGT on the evolution of the human-associated subspecies of *B*. *longum* was found to be considerably high. The effect of homologous recombination on the diversity of housekeeping genes was found to be comparable to the effect of point mutations, similar to the result acquired for *Bacillus* and *Escherichia* [86]. This could be explained at least in part by the abundance of phages and the existence of conjugative plasmids in the populations of humans-associated bifidobacteria. The pan-genome of *B*. *longum* subsp. *longum* (and in some way the pan-genome of the species *B*. *longum*) was found to be open meaning that new orthologous groups of genes could be discovered in every newly sequenced strain. The non-conservative part of the genome was found to be enriched with genes of cell envelope biosynthesis, such as rhamnose biosynthesis clusters and glycosyltransferase genes, reflecting the high individuality of host-microbe and microbe-microbe interaction systems in every strain of *B*. *longum* subsp. *longum*.

An interesting question for further studies is the relative impact of different HGT mechanisms on the evolution of the three subspecies of *B*. *longum*. An important role in the generation of genomic diversity may be played by temperate phages. The genomes used in a present study contained a large number of prophage sequences, but none of these prophages have been isolated in culture and studied experimentally, so the efficiency of phage-related gene transfer in *B*. *longum* still needs to be determined. Another important source of genomic diversity in bifidobactreria could be associated with plasmids, both small cryptic ones and the recently described megaplasmids. In this study, we observed several cases of isolation of fully identical plasmids from far-related strains isolated from the same patient at the same time, suggesting the possibility of fast plasmid transfer between bifidobacteria. An intriguing fact is that the frequency of plasmid-containing strains is distributed unequally among the three subspecies of *B*. *longum*. While plasmids are highly prevalent in *B*. *longum* subsp. *longum*, it seems like plasmids in the other two subspecies are very scarce if not absent at all [87]. Interestingly, the only plasmid-containing strain of *B*. *longum* subsp. *infantis* 157F was reclassified as *B*. *longum* subsp. *longum* in this study. The causes of such difference between the subspecies are unknown, and the impact of this phenomenon on the genomic diversity in *B*. *longum* should be further evaluated.

In this study we described two individual cases of long-term persistence of closely related strains of *B*. *longum* subsp. *longum* in human intestinal microbiota. However, we haven't succeeded in the isolation of fully identical strains from the same individual at different time points. One could hypothesize, that the situation observed can be explained by the high plasticity of intestinal microbiota on the strain level combined with the fine-tuned selection of subsets of the strains highly adapted for each individual host organism. The factors involved in this selection require further intensive studies and can include the host genotype, the repertoire of immune cell receptors, environmental and diet factors, and the individual composition of other microbes in gut microbiota.

## Supporting Information

S1 Fig Multiple alignment of *glmS* gene nucleotide sequences. Black bands represent the positions which are different from the majority in this column. The *glmS* sequence of *B*. *longum* CECT 7347 strain is putatively formed by HGT of gene fragment from *B*. *breve*.
(PNG)

S2 Fig 16s rRNA gene sequences in B. longum. (A) Neighbor-joining phylogenetic tree of 16s rRNA gene sequences. The analysis included fully assembled gene sequences from the genomes studied, and 5 additional sequences from Genbank database. Numbers in nodes represent bootstrap confidence levels. Strain *B*. *longum* subsp. *suis* JDM301, which phylogenetic position based on 16s rRNA contradicts it’s relatedness determined by other approaches, is underlined. (B) Multiple alignment of V6 region of 16S rRNA gene sequences.
(JPG)

S3 Fig Heatmap of average nucleotide identity (ANI) between strains of *B*. *longum*. The tree was inferred by complete linkage hierarchical clustering.
(PNG)

S4 Fig Length distribution of *B*. *longum* genome sequences. Green circles, strains of *B*. *longum* subsp. *longum*; blue diamonds, strains of *B*. *longum* subsp. *infantis*; red triangles, strains of *B*. *longum* subsp. *suis*.
(PNG)

S5 Fig Ribosomal RNA operons in the complete genomes of *B*. *longum*. (a) Scheme of the positions of three rRNA-containing loci in the genome of *B*. *longum* subsp. *longum* DJO10A. Their positions are similar for all other strains, excluding ATCC 15697, which have the large inverted region, including locus 3 (b) The composition of the simplest rRNA operon of *B*. *longum*. (c) The structure of the three rRNA loci among the complete genomes of *B*. *longum* strains. The locus 1 in the strain 157F contains an insertion of a IS21 family mobile genetic element, containing transposase gene with a frameshift mutation (shown in yellow).
(PNG)

S6 Fig Quantities of ortholog groups, belonging to different functional classes, in the “average” genome of B. *longum* subsp. *longum* strain and the pan-genome of this subspecies. Three classes (“RNA processing and modification”, “Cell motility” and “Secondary metabolites biosynthesis, transport and catabolism”), representing <0.5% each are not shown.
(PNG)

S7 Fig Average quantities of ortholog groups, belonging to different functional classes, in the genomes of different subspecies of B. *longum*. Error bars represent the standard deviations.
(PNG)

S8 Fig Length distribution of open reading frames in ortholog groups of *B*. *longum* subsp. *longum* pan-genome, which were classified and not classified using COG database. (PNG)

S9 Fig Neighbor-joining phylogenetic tree of pKJ36/pB44 family plasmids. Numbers in nodes represent bootstrap confidence levels.
(PNG)

S10 Fig Spacers within the CRISPR-Cas systems of *B*. *longum*. Each spacer is represented by a colored box. The colors of the boxes represent the locus they appeared for the first time in the direction upside down. Each locus is represented in the putative direction of transcription (from left to right). The symbols in the scheme: #, the insertion sequence; *, the place of system disruption by the putative translocation;?, the non-sequenced region, containing disruption of an unknown structure.
(PNG)

S1 File ‘Core addition’ proteins of *B*. *longum* subsp. *longum*. Sequences were obtained from the genome of *B*. *longum* subsp. *longum* 12_1_47BFAA.
(TXT)

S2 File ‘Core addition’ proteins of *B*. *longum* subsp. *infantis*. Sequences were obtained from the genome of *B*. *longum* subsp. *infantis* ATCC 15697^T^.
(TXT)

S3 File ‘Core addition’ proteins of *B*. *longum* subsp. *suis*. Sequences were obtained from the genome of *B*. *longum* subsp. *suis* AGR2137.
(TXT)

S1 Table Housekeeping genes used for phylogenetic analysis. (PDF)

S2 Table Genomes of *B*. *longum* analyzed in this study. (PDF)
