# Supplementary figures and images for: Intraspecies Genomic Diversity and Long-Term Persistence of Bifidobacterium longum
Source: PLoS One. 2015 Aug 14;10(8):e0135658. doi: 10.1371/journal.pone.0135658 (PMC4537262; doi:10.1371/journal.pone.0135658)

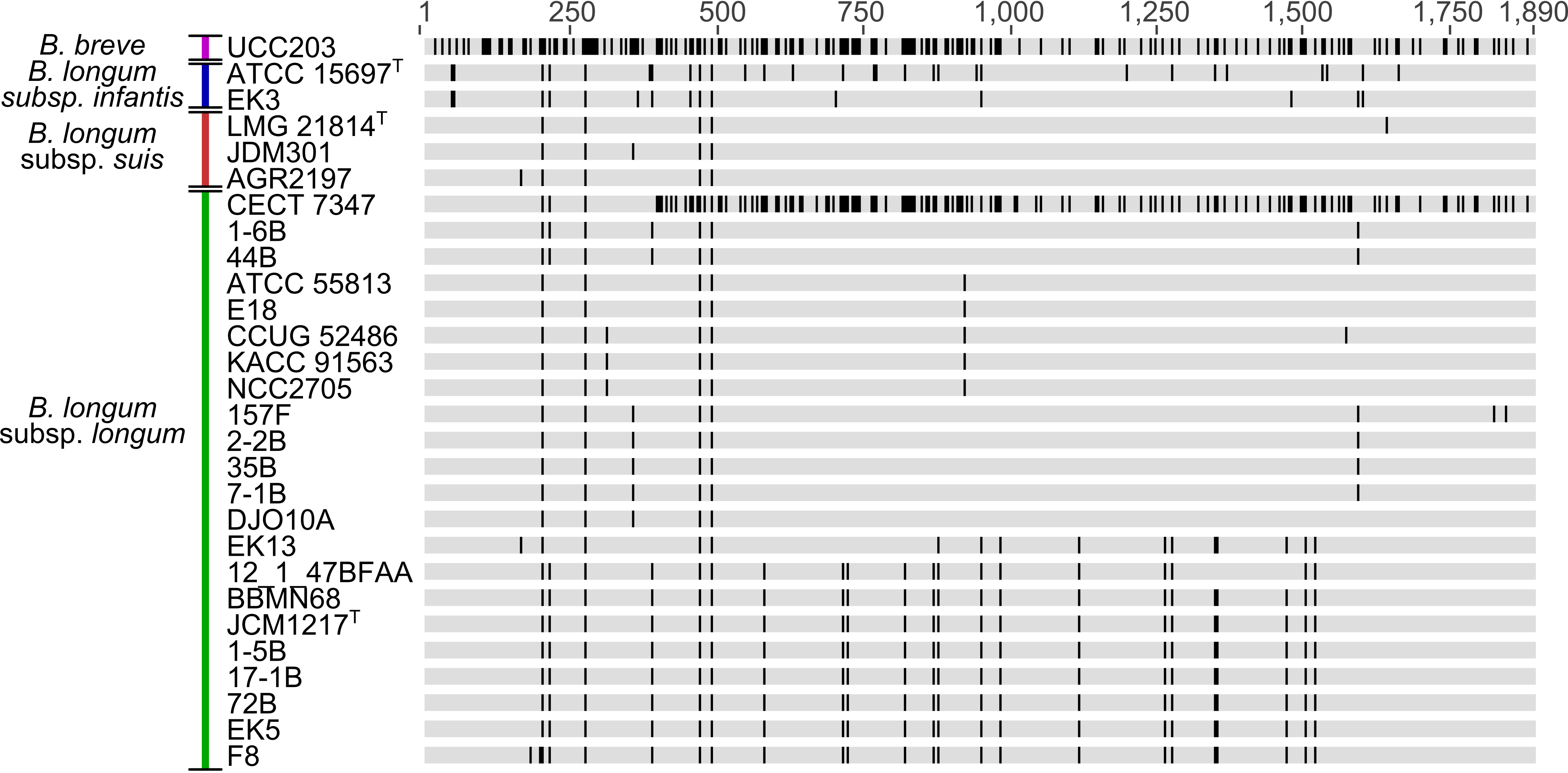

Supplement: S1 Fig — Black bands represent the positions which are different from the majority in this column. The glmS sequence of B. longum CECT 7347 strain is putatively formed by HGT of gene fragment from B. breve. (PNG) [file pone.0135658.s001.png]

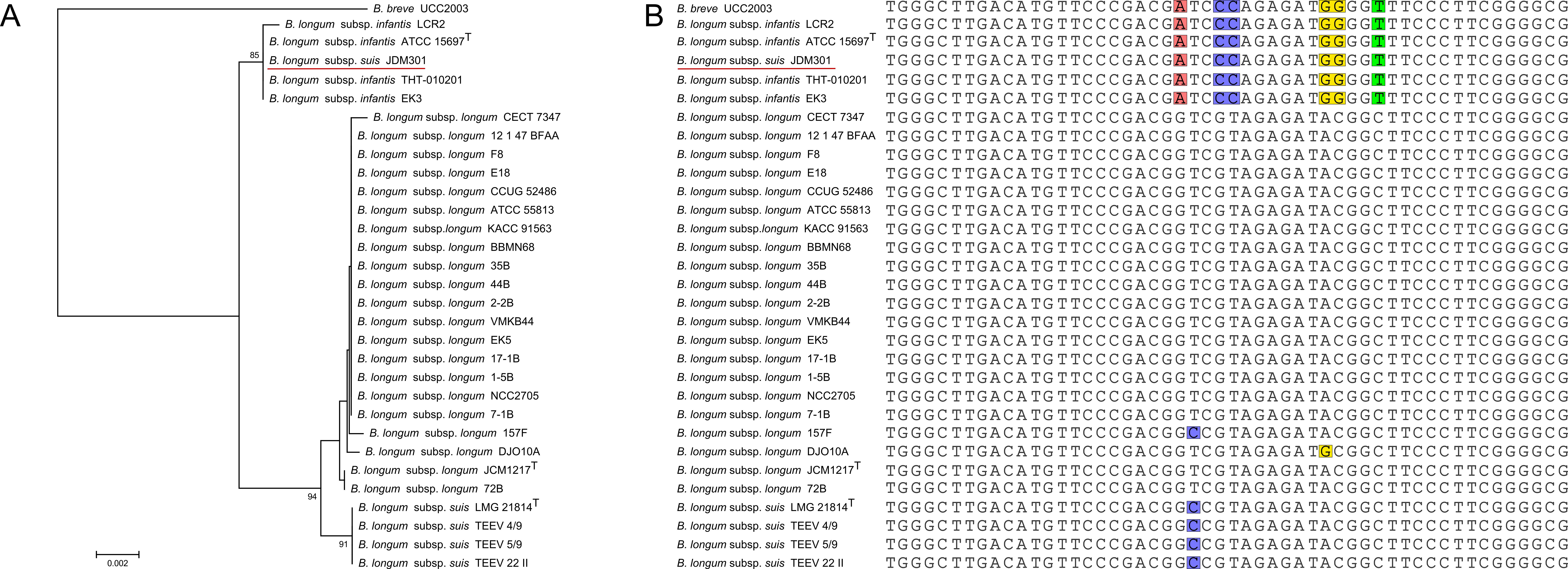

Supplement: S2 Fig — (A) Neighbor-joining phylogenetic tree of 16s rRNA gene sequences. The analysis included fully assembled gene sequences from the genomes studied, and 5 additional sequences from Genbank database. Numbers in nodes represent bootstrap confidence levels. Strain B. longum subsp. suis JDM301, which phylogenetic position based on 16s rRNA contradicts it’s relatedness determined by other approaches, is underlined. (B) Multiple alignment of V6 region of 16S rRNA gene sequences. (JPG) [file pone.0135658.s002.jpg]

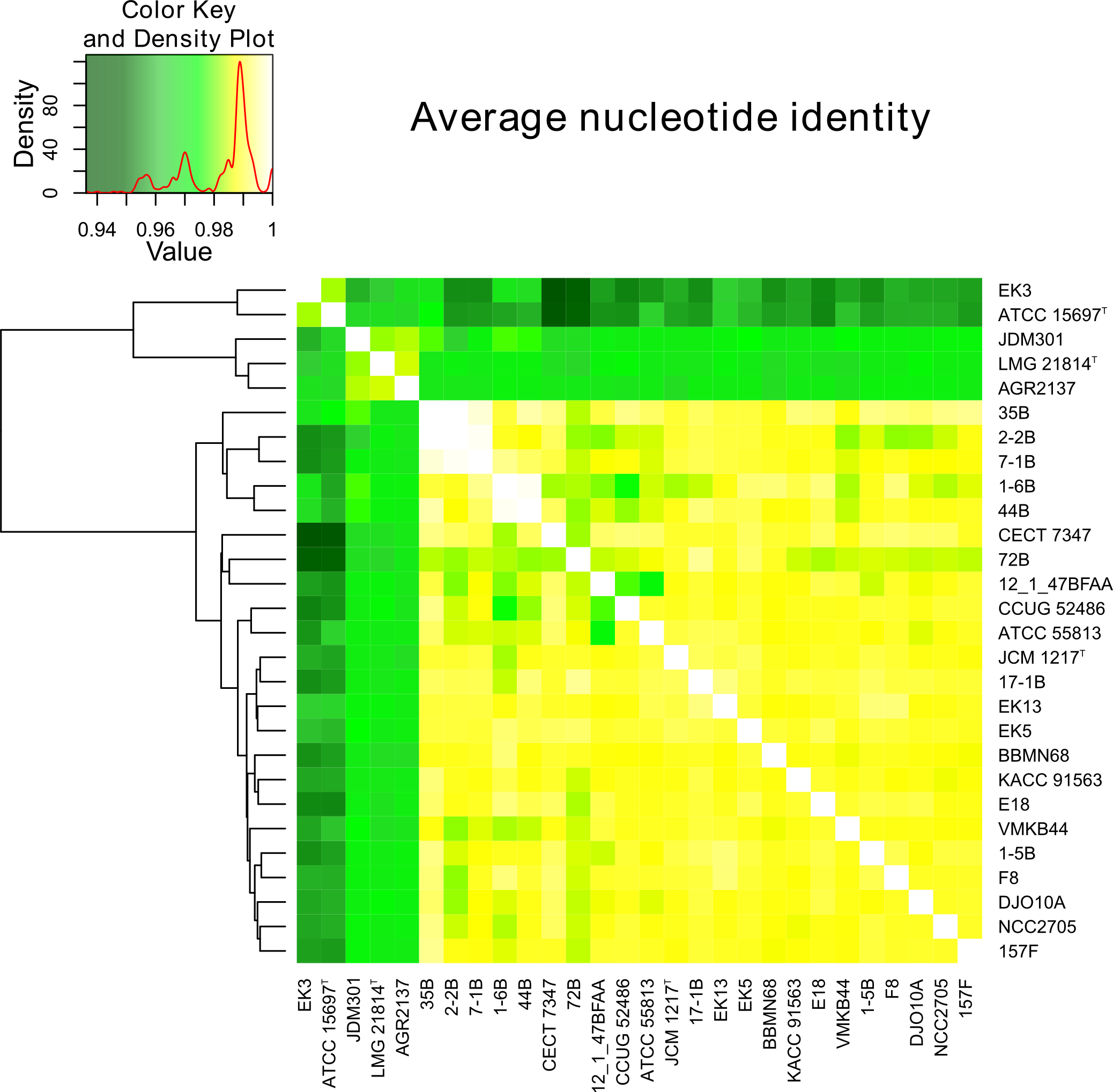

Supplement: S3 Fig — The tree was inferred by complete linkage hierarchical clustering. (PNG) [file pone.0135658.s003.png]

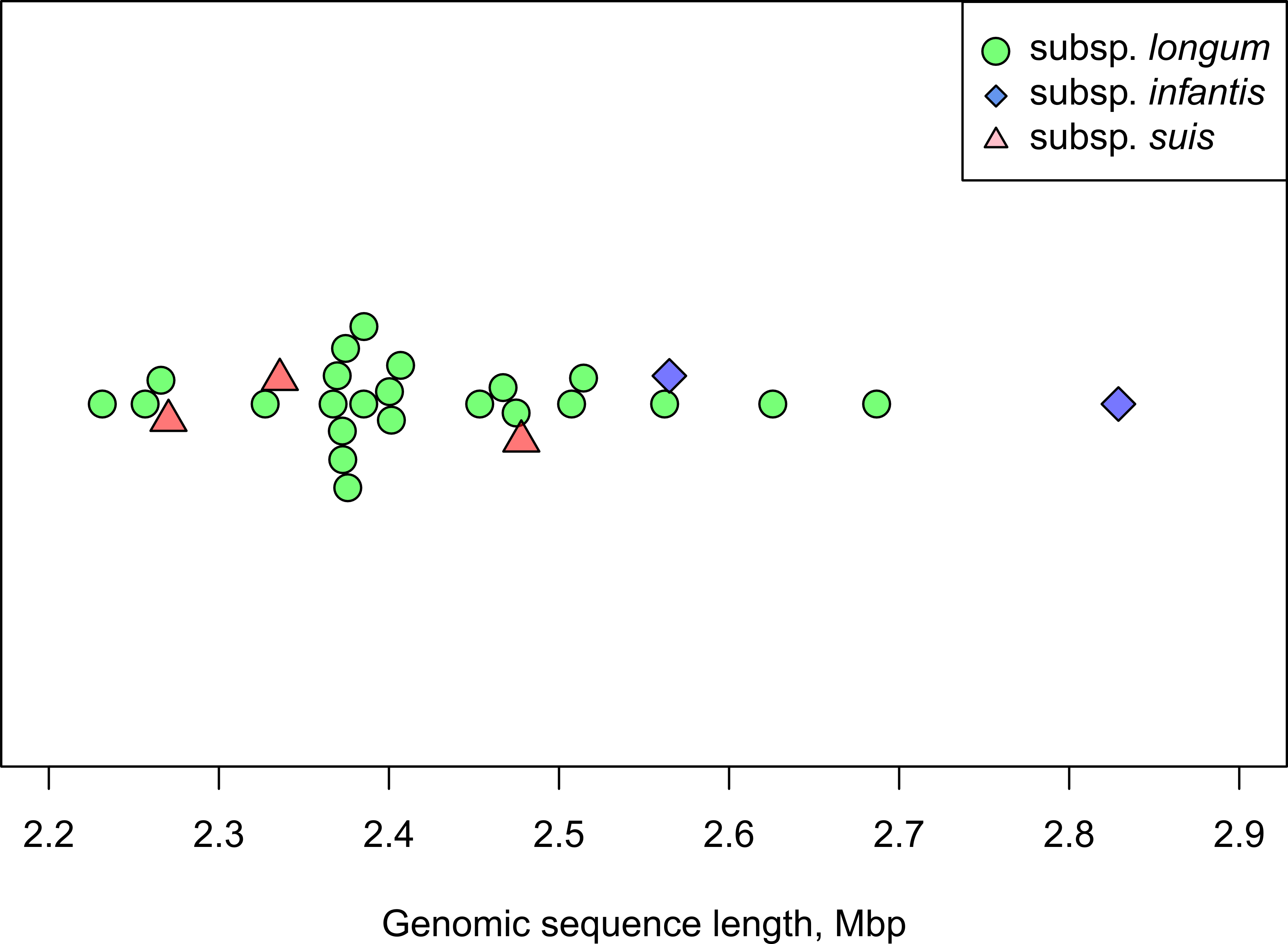

Supplement: S4 Fig — Green circles, strains of B. longum subsp. longum; blue diamonds, strains of B. longum subsp. infantis; red triangles, strains of B. longum subsp. suis. (PNG) [file pone.0135658.s004.png]

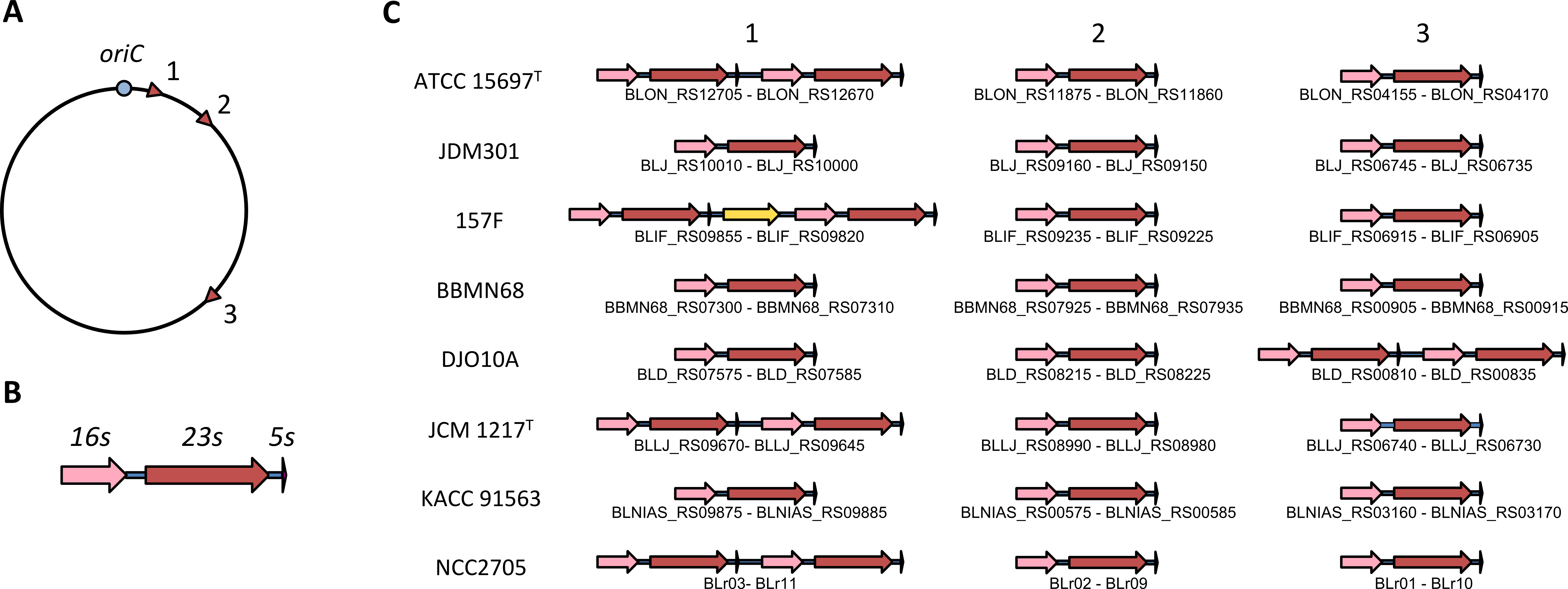

Supplement: S5 Fig — (a) Scheme of the positions of three rRNA-containing loci in the genome of B. longum subsp. longum DJO10A. Their positions are similar for all other strains, excluding ATCC 15697, which have the large inverted region, including locus 3 (b) The composition of the simplest rRNA operon of B. longum. (c) The structure of the three rRNA loci among the complete genomes of B. longum strains. The locus 1 in the strain 157F contains an insertion of a IS21 family mobile genetic element, containing transposase gene with a frameshift mutation (shown in yellow). (PNG) [file pone.0135658.s005.png]

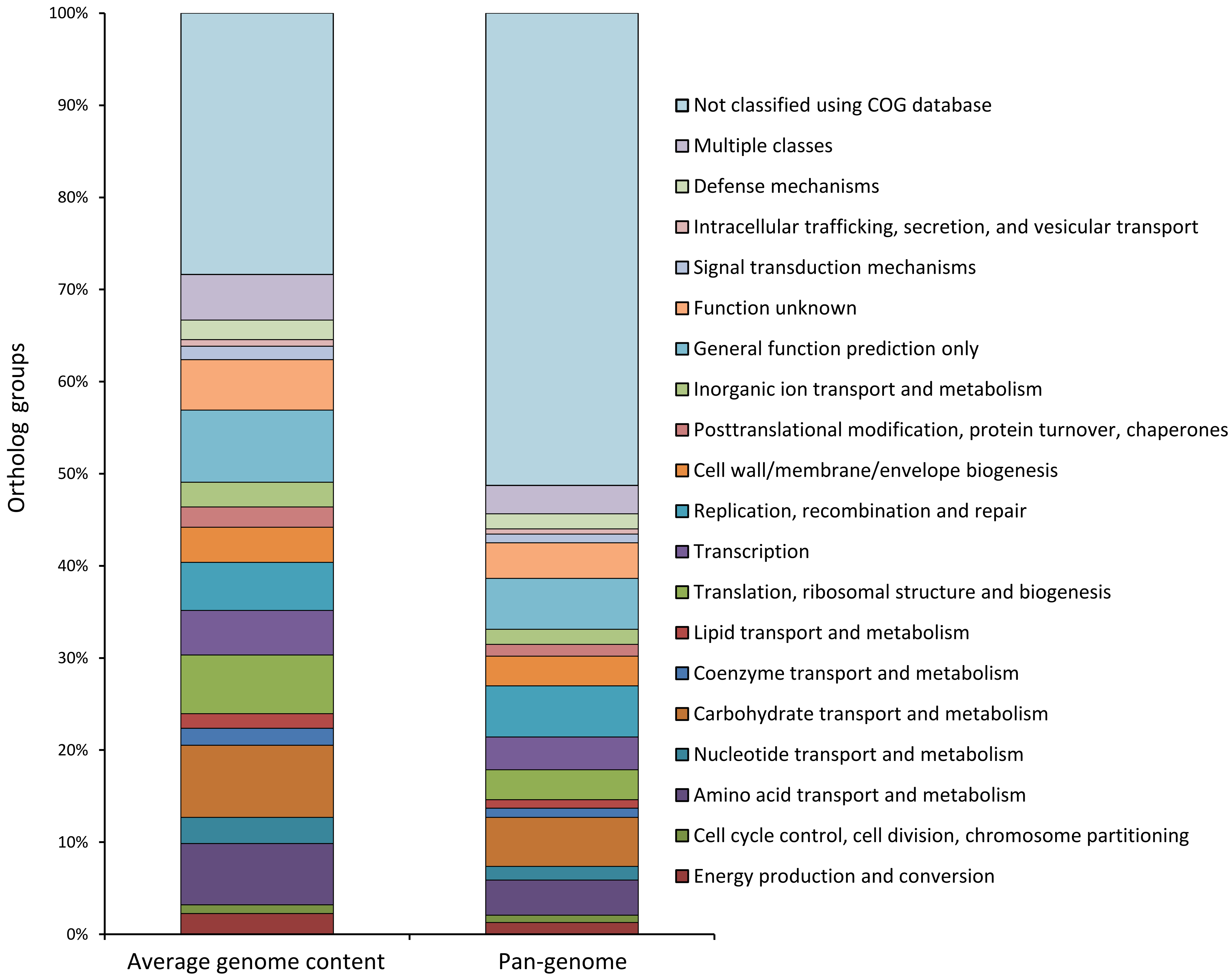

Supplement: S6 Fig — Three classes (“RNA processing and modification”, “Cell motility” and “Secondary metabolites biosynthesis, transport and catabolism”), representing <0.5% each are not shown. (PNG) [file pone.0135658.s006.png]

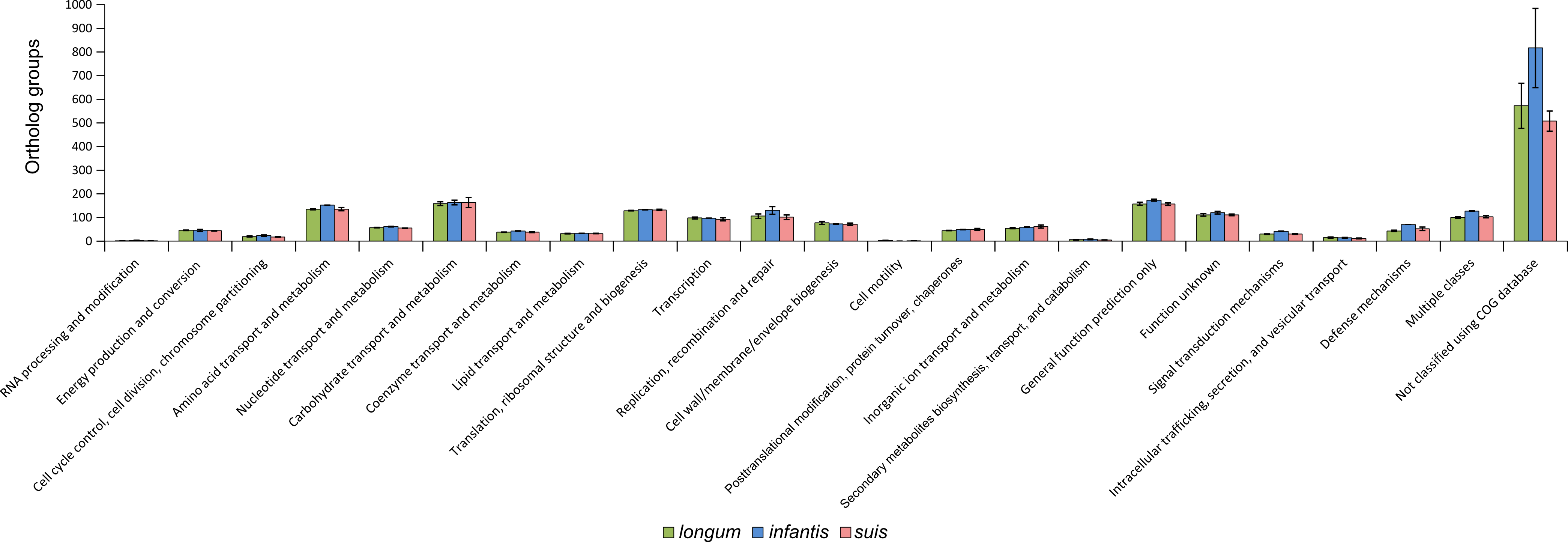

Supplement: S7 Fig — Error bars represent the standard deviations. (PNG) [file pone.0135658.s007.png]

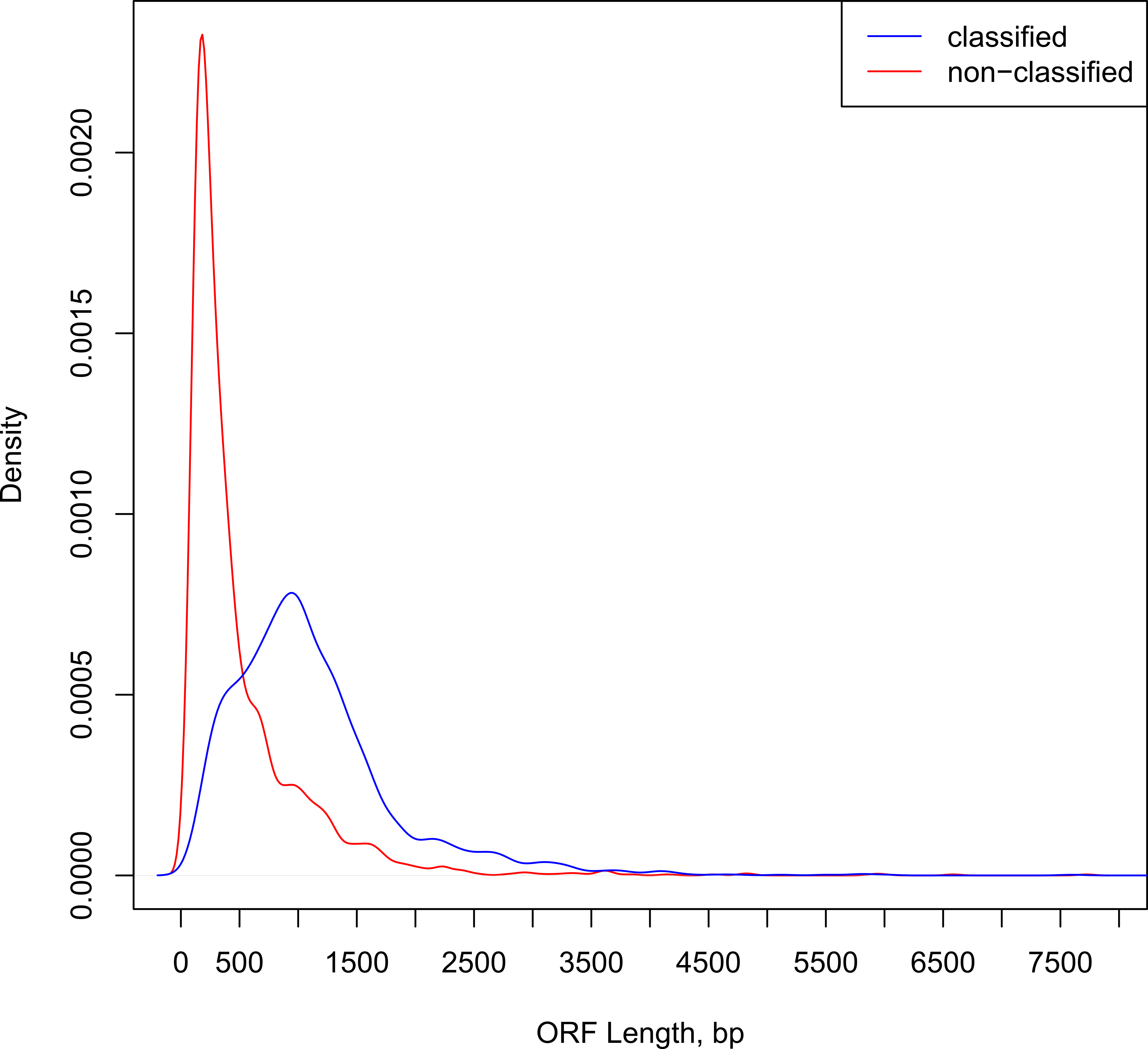

Supplement: S8 Fig — (PNG) [file pone.0135658.s008.png]

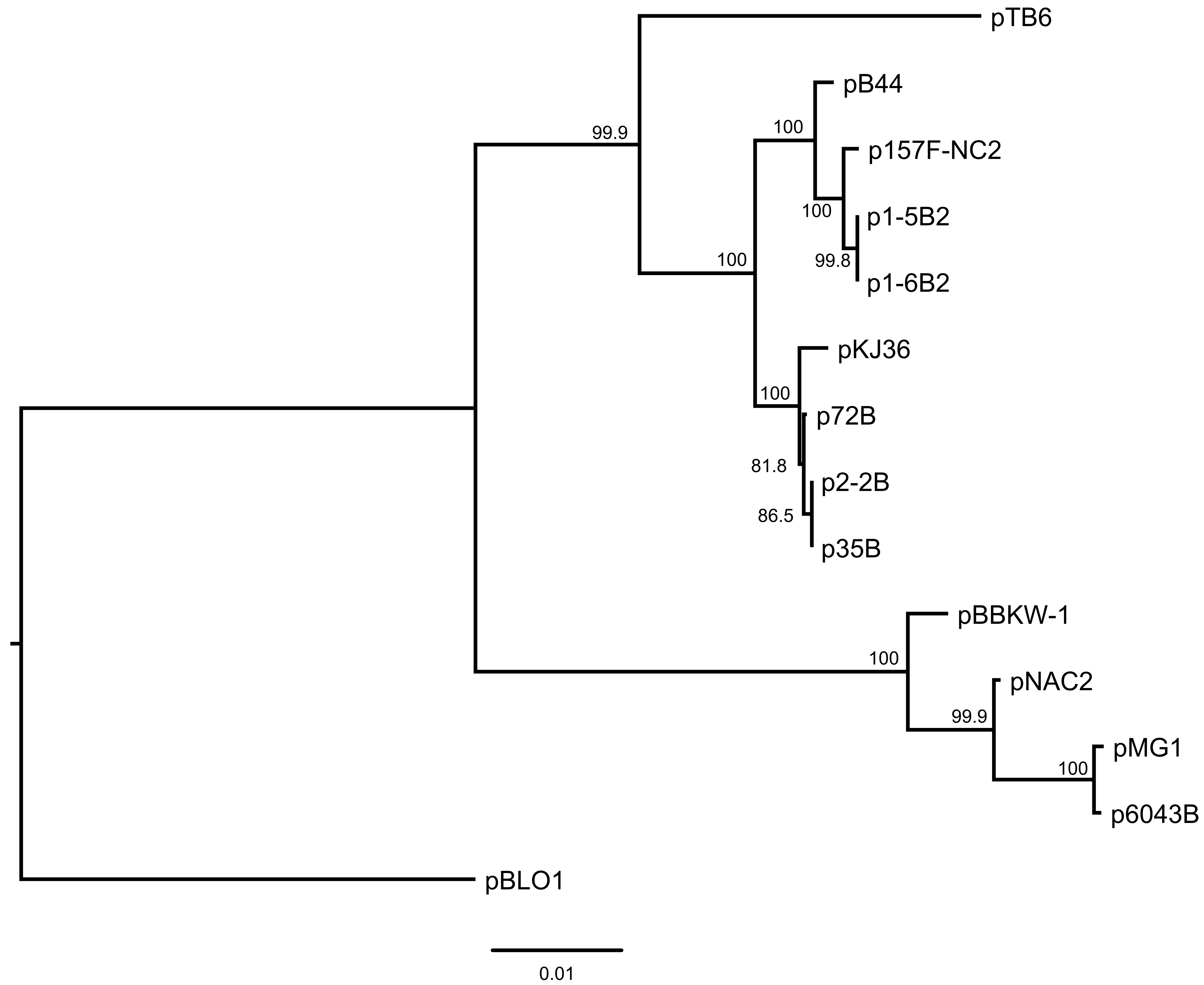

Supplement: S9 Fig — Numbers in nodes represent bootstrap confidence levels. (PNG) [file pone.0135658.s009.png]

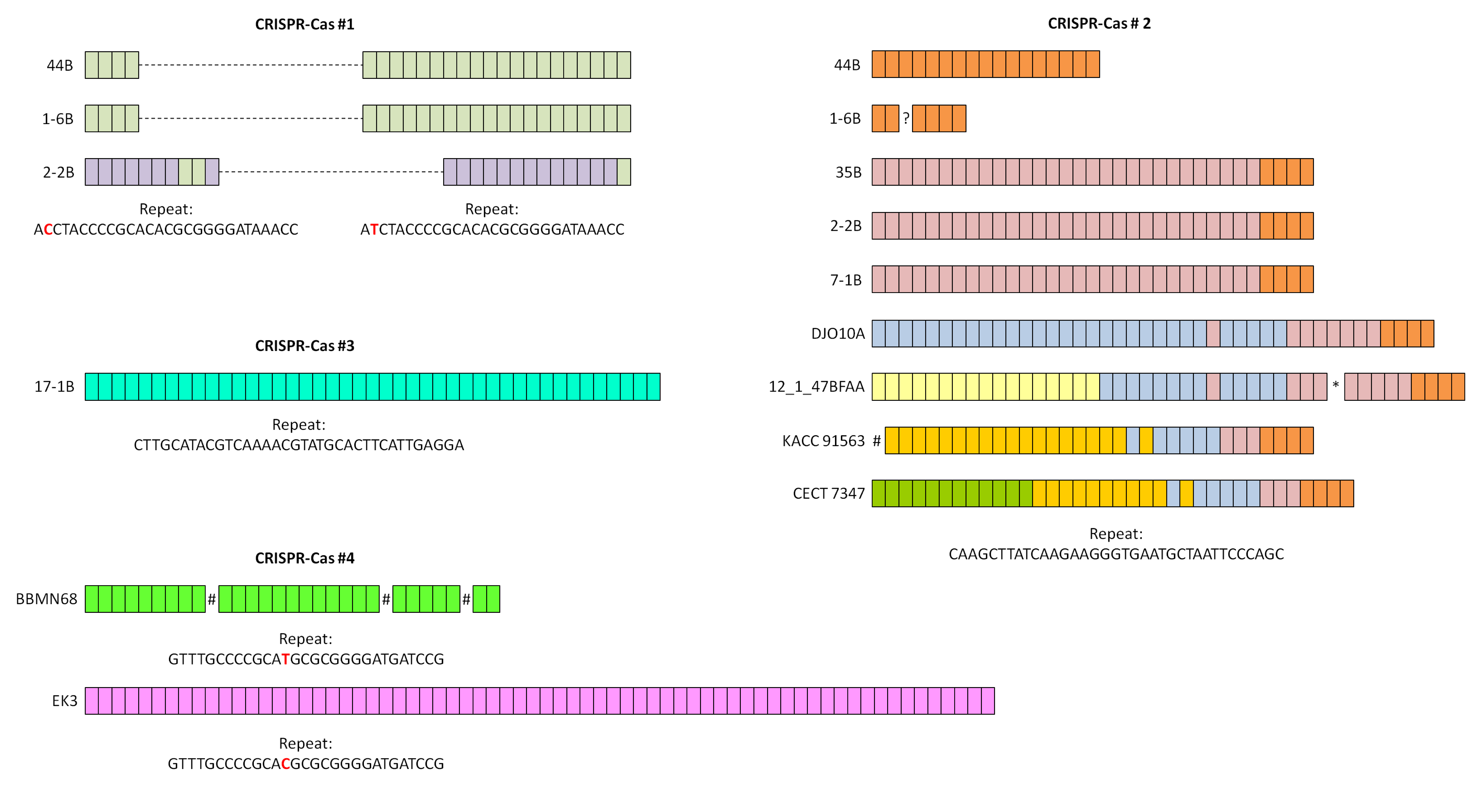

Supplement: S10 Fig — Each spacer is represented by a colored box. The colors of the boxes represent the locus they appeared for the first time in the direction upside down. Each locus is represented in the putative direction of transcription (from left to right). The symbols in the scheme: #, the insertion sequence; *, the place of system disruption by the putative translocation;?, the non-sequenced region, containing disruption of an unknown structure. (PNG) [file pone.0135658.s010.png]
