# Supplementary material for: Intraspecies Genomic Diversity and Long-Term Persistence of Bifidobacterium longum
Source: PLoS One. 2015 Aug 14;10(8):e0135658. doi: 10.1371/journal.pone.0135658 (PMC4537262; doi:10.1371/journal.pone.0135658)
Supplement: S1 Table — (PDF) [file pone.0135658.s014.pdf]

Table S1. Housekeeping genes used for phylogenetic analysis.

Gene abbr.	Product
ackA	Acetate kinase
aspS	Aspartyl-tRNA synthetase
cysS	Cysteinyl-tRNAsynthetase
ddl	D-alanyl-D-alanine synthetase A
dnaK	chaperone DnaK
dnaX	DNA polymerase III subunits γ and τ
eno	Enolase
frr	Ribosome recycling factor
ftsZ	Cell division protein FtsZ
fusA	Elongation factor G
glmS	Glucosamine-fructose-6-phosphate aminotransferase
groL	Chaperonin GroEL
groS	Co- chaperonin GroES
gyrA	DNA gyrase subunit A
holA	DNA polymerase III subunit δ
ldh2	Lactate dehydrogenase
ligA	DNA ligase
murA	UDP-N-acetylglucosamine 1-carboxyvinyltransferase
murC	UDP-N-acetylmuramate-alanine ligase
murE	UDP-N-acetylmuramoylalanyl-D-glutamate-2, 6-diaminopimelate ligase
murI	Glutamate racemase
pgm	Phosphoglucomutase
polA	DNA polymerase I
ppa	Inorganic pyrophosphatase
prfA	Protein releasing factor
proS	Prolyl-tRNAsynthetase
purA	Adenylosuccinate synthetase
purB	Adenylosuccinate lyase
purF	Glutamine phosphoribosylpyrophosphate amidotransferase
purH	Bifunctional 5'-phosphoribosyl 5-aminoimidazole-4-carboxamide transformylase-IMP cyclohydrolase
pyrK	Dihydroorotate dehydrogenase electron transfer subunit
recA	Recombinase A
rho	Transcription termination factor Rho
rplY	50S ribosomal protein L25
rpoA	DNA-directed RNA polymerase subunit α
rpoC	DNA-directed RNA polymerase subunit β’
rpoD	RNA polymerase σ factor
rpoZ	DNA-directed RNA polymerase subunit ω
rpsO	30S ribosomal protein S15
tig	Trigger factor
tkt	Transketolase
topA	DNA topoisomerase I
trpS	Tryptophanyl-tRNA synthetase
