# Supplementary material for: Intraspecies Genomic Diversity and Long-Term Persistence of Bifidobacterium longum
Source: PLoS One. 2015 Aug 14;10(8):e0135658. doi: 10.1371/journal.pone.0135658 (PMC4537262; doi:10.1371/journal.pone.0135658)
Supplement: S2 Table — (PDF) [file pone.0135658.s015.pdf]

Strain name	GenBank accession number	Genome sequence status	Sequencing technology	Coverage	N50, bp	Sequence length, Mbp	Subspecies identified in this study
ATCC 15697[T]	NC_011593	Complete	Sanger	8x	2,832,748	2.83	*infantis*
EK3	JNWC00000000	45 contigs	Illumina	1161x	184,521	2.56	*infantis*
AGR2137	ATWX00000000	49 contigs	Illumina	Not provided	79,605	2.27	*suis*
JDM301	NC_014169	Complete	454	16x	2,477,838	2.48	*suis*
LMG 21814[T]	JGZA00000000	36 contigs	Ion Torrent	71x	96,45	2.34	*suis*
12_1_47BFAA	NZ_ADCN00000000	86 contigs	454	34x	60,093	2.40	*longum*
157F	NC_015052	Complete	Sanger	7.8x	2,400,312	2.40	*longum*
1-5B	JNVX00000000	28 contigs	Illumina	3037x	286,86	2.37	*longum*
17-1B	JNVZ00000000	23 contigs	Illumina	2001x	247,559	2.47	*longum*
1-6B	AJTF00000000	171 contigs	454	14x	58,499	2.69	*longum*
2-2B	AJTJ00000000	141 contigs	454	11x	39,901	2.63	*longum*
35B	AJTI00000000	131 contigs	454	11x	57,033	2.51	*longum*
44B	AJTM00000000	62 contigs	454	25x	90,207	2.56	*longum*
72B	JNWA00000000	39 contigs	Illumina	1059x	186,419	2.37	*longum*
7-1B	JNVY00000000	37 contigs	Illumina	2814x	435,415	2.41	*longum*
ATCC 55813	ACHI00000000	140 contigs	454	53x	137,057	2.37	*longum*
BBMN68	NC_014656	Complete	454	38x	2,265,943	2.27	*longum*
CCUG 52486	NZ_ABQQ00000000	55 contigs	454	34x	171,444	2.45	*longum*
CECT 7347	NZ_CALH00000000	128 contigs	454	32x	39,694	2.33	*longum*
DJO10A	NC_010816	Complete	Sanger	9.2x	2,375,792	2.38	*longum*
E18	AUYD00000000	7 contigs	454	19.6x	1,251,618	2.37	*longum*
F8	NC_021008	21 contigs	454	45x	230,054	2.37	*longum*
EK5	JNWC00000000	35 contigs	Illumina	1417x	254,81	2.23	*longum*
EK13	JNWD00000000	48 contigs	Illumina	1337x	303,761	2.47	*longum*
JCM 1217[T]	NC_015067	Complete	Sanger	7.6x	2,385,164	2.39	*longum*
KACC 91563	NC_017221	Complete	454	222x	2,385,301	2.39	*longum*
NCC2705	NC_004307	Complete	Sanger	8x	2,256,640	2.26	*longum*
VMKB44	JRWN00000000	36 contigs	Illumina	913x	177,067	2.51	*longum*
